# Enhanced secretion of promyogenic exosomes by quiescent muscle cells

**DOI:** 10.3389/fcell.2024.1381357

**Published:** 2024-07-23

**Authors:** Prabhavathy Devan, Ananga Ghosh, Pallavi Rao T., Swasti Raychaudhuri, Harikrishna Adicherla, Himadri Devanshi, Pallavi Kshetrapal, Jyotsna Dhawan

**Affiliations:** ^1^ CSIR-Centre for Cellular and Molecular Biology, Hyderabad-500007, India; ^2^ Academy of Scientific and Innovative Research (AcSIR), Ghaziabad-201002, India; ^3^ Perinatal Biology Lab, Maternal Child Health Department, Translational Health Science and Technology Institute, NCR Biotech Science Cluster, Faridabad-121001, India

**Keywords:** quiescence, myoblast, G_0_, SEV, exosomes, KIBRA, Wnt, differentiation

## Abstract

Signaling interactions are important during skeletal muscle regeneration, where muscle cells in distinct states (quiescent, reactivated, proliferating and differentiated) must coordinate their response to injury. Here, we probed the role of secreted small extracellular vesicles (sEV/exosomes) using a culture model of physiologically relevant cell states seen in muscle regeneration. Unexpectedly, G_0_ myoblasts exhibited enhanced secretion of sEV (∼150 nm) displaying exosome markers (Alix, TSG101, flotillin-1, and CD9), and increased expression of Kibra, a regulator of exosome biogenesis. Perturbation of Kibra levels confirmed a role in controlling sEV secretion rates. Purified sEVs displayed a common exosome marker-enriched proteome in all muscle cell states, as well as state-specific proteins. Exosomes derived from G_0_ cells showed an antioxidant signature, and were most strongly internalized by differentiated myotubes. Functionally, donor exosomes from all muscle cell states could activate an integrated Wnt reporter in target cells, but only G_0_-derived exosomes could induce myogenic differentiation in proliferating cells. Taken together, we provide evidence that quiescence in muscle cells is accompanied by enhanced secretion of exosomes with distinct uptake, cargo and signal activating features. Our study suggests the novel possibility that quiescent muscle stem cells *in vivo* may play a previously under-appreciated signaling role during muscle homeostasis.

## Introduction

Inter-cellular communication plays a vital role in coordinating tissue form and function. In addition to individual secreted proteins such as cytokines, mitogens and morphogens, growing evidence points to a role for complex signals delivered by small extracellular vesicles (sEV) in the size range of 30–150 nm (Corona et al., 2023). Unlike other classes of sEV that emerge via budding from the plasma membrane, the “exosome” subclass is derived by endocytic generation of multivesicular bodies (MVB) that fuse with the plasma membrane (PM) releasing intraluminal vesicles (ILV) as sEV/exosomes (Gurung et al., 2021). Exosomes ferry a diverse cargo including membrane-anchored receptors/ligands, intra-cellular proteins, metabolites, RNA and DNA, constituting a high potential for modulating target cell behaviour (Vyas and Dhawan, 2017; Zhang et al., 2019). Exosomes have emerged as potent mediators of short-range signalling between different cell types (McGough and Vincent, 2016), as well as long-range signalling between organs (Rios-Colon et al., 2020; Lassen et al., 2021). The limited diffusibility of factors such as Wnt, Hh and Notch ligands due to lipid modification or membrane anchoring has triggered interest in exosomes as carriers of these signals during development (Vyas et al., 2014; McGough and Vincent, 2016). Profiling of nucleic acids in serum exosomes is being increasingly used for diagnosis (Yao et al., 2022), while engineered exosomes have a variety of therapeutic applications (Pan et al., 2023).

Exosomal signaling between different cell types such as those of the circulating immune system and tissue-resident cells is known to play a role in development and disease (Zou et al., 2021). However, little is known about the exosomal output of a given cell type in different states, which may be important for cellular cross-talk during regeneration, where a spectrum of progenitor and differentiating cells of the same tissue origin must coordinate the response to injury leading to repair. For example, in adult skeletal muscle at homeostasis, the bulk of tissue is comprised of differentiated multinucleated muscle fibers, with a small population of dormant muscle stem cells (MuSCs) harbored in a myofiber niche that is influenced by endothelial cells, fibroblasts, neurons and macrophages (Mounier et al., 2011). In response to myofiber injury, quiescent MuSCs are activated to divide; most of the progeny differentiate to regenerate damaged myofibers, while a small sub-population undergoes self-renewal to replenish the quiescent stem cell compartment (Brunet et al., 2023).

The major signaling pathways including Wnt, Notch, Hh, TGF-β and insulin/IGF control different phases in muscle regeneration (Demonbreun and McNally, 2017). In particular, Wnt factors secreted by myoblasts influence early myotube differentiation (Brack et al., 2008; von Maltzahn et al., 2012), while Notch ligands on cells adjacent to quiescent MuSCs promote self-renewal (Conboy and Rando, 2002; Baghdadi et al., 2018; Gioftsidi et al., 2022). Differentiated muscle secretes an array of signals including mitogens (IGFs), and myokines (myostatin, FGF 21, LIF, IGF-1) (Leuchtmann et al., 2021).

The role of EVs in muscle tissue is an active area of investigation (Bittel and Jaiswal, 2019). For example, exosome-mediated signaling from muscle cells to fibroblasts controls ECM deposition during regeneration (Fry et al., 2017; Zanotti et al., 2018). Further, muscle cells isolated from ALS patients secrete neurotoxic exosomes *in vitro* (Le Gall et al., 2022). However, little is known about the relative contribution of muscle cells in different states to the signaling milieu during periods of tissue reorganization following muscle damage. External signals that activate quiescent MuSCs during regeneration are well studied (Gioftsidi et al., 2022; Loreti and Sacco, 2022), but signals emanating from quiescent MuSCs during homeostasis are poorly explored.

Few tissue-specific markers of sEVs are known (Deng and Miller, 2019), precluding the unambiguous identification and isolation of sEV/exosomes produced by specific cell types/cell states *in vivo*. To address the question of whether muscle cell states observed during regeneration differ in their exosomal output and impact, we have used a cultured myoblast system to model these distinct cell states: differentiated myofibers, as well as quiescent, reactivated and proliferating myoblasts (Arora et al., 2017). Earlier studies have documented exosome profiles of proliferating and differentiated muscle cells (Forterre et al., 2014), but exosome production and signaling by quiescent myoblasts have not been reported.

Here we report that exosomal output, uptake, cargo and signaling capability are modulated during cell state changes within a single cell type. Unexpectedly, we find that quiescent myoblasts secrete higher numbers of exosomes with unique promyogenic signaling properties. Thus, dormant cells can serve as the source of signals rather than only serving as targets for activating signals. These findings have interesting implications for intercellular communication during muscle homeostasis and regeneration, when stem cells undertake different fates to repair damaged tissue.

## Results

In order to probe the role of sEV in intercellular signaling as skeletal muscle cells enter different states, we used an established C2C12 myoblast culture model (Yaffe, 1968; Blau et al., 1983; Gunning et al., 1983). This culture model recapitulates cellular states found in normal and damaged skeletal muscle *in vivo* (Milasincic et al., 1996; Dhawan and Helfman, 2004; Arora et al., 2017) (Supplementary Figure S1A, B). We used established assays and markers to confirm four clearly distinguishable cellular states (Supplementary Figure S1C–S1E) [exponentially proliferating myoblasts (MB), resting myoblasts 24 h after induction of quiescence (G_0_), reactivated myoblasts 24 h after release from G_0_ (R24), and differentiated myotubes 72 h after induction of myogenesis (MT)].

### Quiescent myoblasts secrete more sEVs than proliferating cells, reverting to lower secretion rate after cell cycle reactivation

**FIGURE 1:**
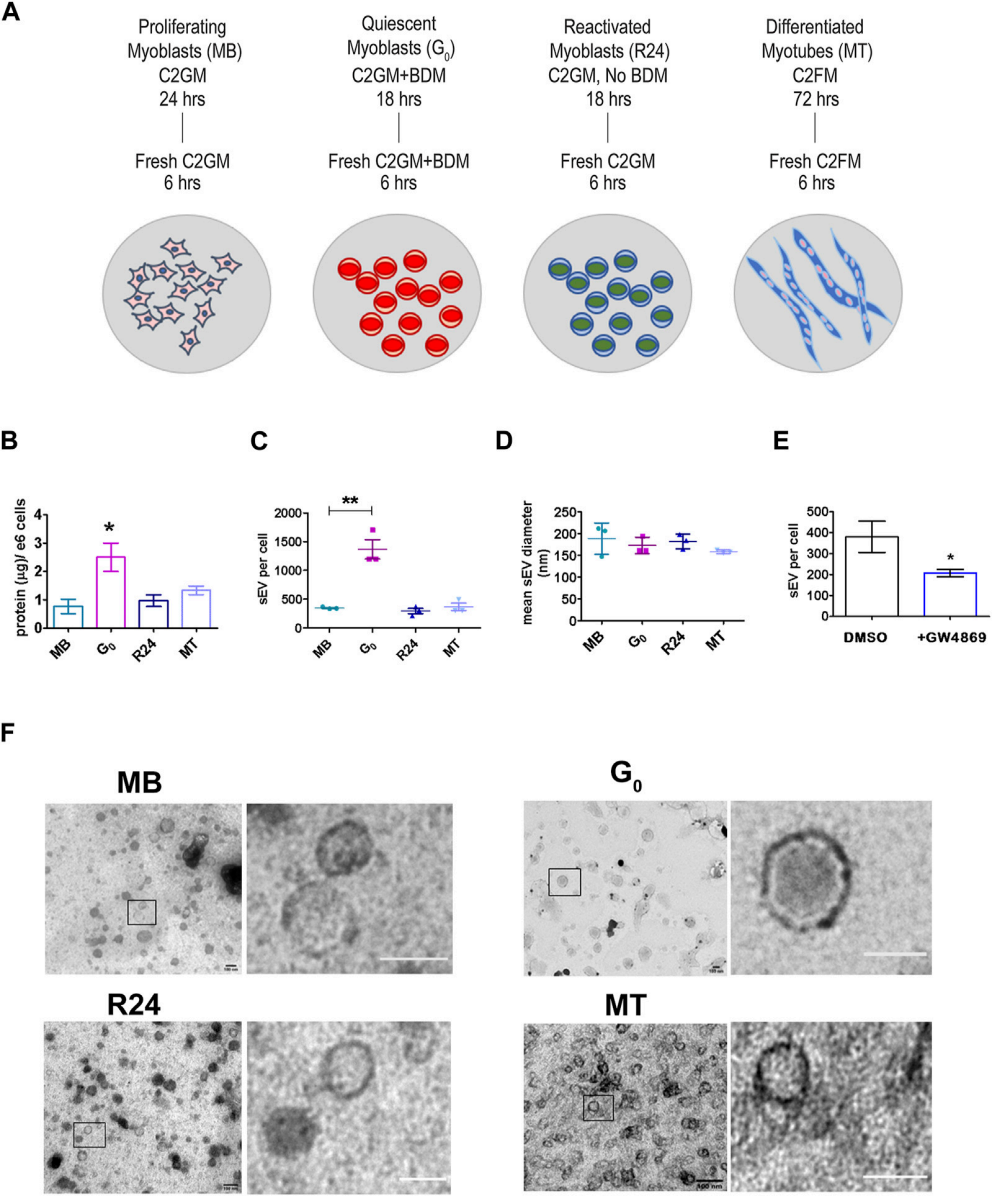
*Quiescent cells secrete more sEVs than other cellular states* **(A)**. Schematic showing the generation of muscle cells states generated *in vitro* using cultured mouse myoblasts to recapitulate the *in vivo* states: Sub-confluent cultures of asynchronously proliferating myoblasts (MB) are treated with 30 mM BDM (myosin II inhibitor) for 24 h in growth medium (GM, 20% FBS) to generate synchronized cultures of quiescent myoblasts (G_0_). After 24 h, GM + BDM is replaced with fresh GM (without BDM) for 24 h to generate synchronously reactivated myoblasts (R24). To generate differentiated myotubes (MT), dense cultures of MB (90% confluency) are incubated in differentiation medium (DM, 2% HS) for 3 days during which time they fuse to form multinucleated myotubes (MT) (see Supplementary Figure S1 for details). Conditioned media was collected for 6 h in fresh EPM appropriate to each state. **(B)**. Quantitation of total protein in purified sEV fraction isolated from CM of proliferation (MB), quiescence (G_0_), reactivation for 24 h (R24) and 3 days of differentiation (MT). Significant increase in yield of sEV protein per 10^6^ cells per 6 h in G_0_. Values represent mean ± SEM n = 5, *p ≤ 0.05 compared against MB. **(C)**. Nanoparticle Tracking Analysis (NTA) of sEVs isolated from 6 h CM from each state shown in **(A)**. Number of sEV secreted per cell by 10^6^ cells in each state in 6 h: G_0_ cells secrete substantially higher numbers of exosomes. Values represent mean ± SEM n = 3 within the same cellular condition. **(D)**. Mean diameter of sEVs as determined by NTA. **(E)**. sEV secretion is suppressed by GW4869, an inhibitor of exosome biogenesis. **(F)**. Representative images from TEM analysis of whole mounts of purified sEV isolated by ultracentrifugation from conditioned media of MB, G_0_, R24 and MT states after negative staining with uranyl acetate. n = 3. All images showed the presence of numerous intact vesicles with heterogeneous size distribution and density as predicted from NTA analysis (Figure 1D) and sucrose gradient centrifugation (Supplementary Figure S3C) respectively. Boxed area in each panel is zoomed in right panel to highlight individual vesicles; scale bar in magnified panels: MB and G_0_ −100 nm; R24–75 nm; MT—50 nm.

sEVs were isolated from equal number of cells in each of the different muscle cell states after medium was conditioned for an equal time interval (6 h, Figure 1A). Given that the different cell states require distinct culture conditions, appropriate exosome production medium (EPM) depleted of serum exosomes was generated for each culture state (see Methods) and tested to ensure equivalent growth promoting activity as control media (Supplementary Figure S2). Thus, our protocol ensures collection of sEVs secreted in a defined time by a defined number of cells in distinct physiologically relevant stable states.

Unexpectedly, G_0_ cells secreted substantially more sEVs than an equivalent number of proliferating MB, as evidenced by the yield of total protein in exosome fraction (Figure 1B) as well as by nanoparticle tracking analysis (NTA) (Figure 1C). This enhanced secretion was reversible: 24 h after G_0_ cells were induced to re-enter the cell cycle (R24), sEV secretion returned to the levels seen in asynchronously proliferating MB. Secretion rate in terminally arrested MT was also similar to that in MB. Thus, despite a lower overall metabolic rate (Valcourt et al., 2012), G_0_ cells unexpectedly showed enhanced sEV secretion compared to other cellular states. In all four states, particle size (mean diameter) estimated by NTA (140–180 nm, Figure 1D, Supplementary Figure S3A,B) was consistent with that reported for exosomes (Dehghani et al., 2020).

To confirm the composition of the isolated fraction, GW4869 (2.5 µM) an inhibitor of neutral sphingomyelinase was used to directly inhibit exosome biogenesis, leading to ∼50% reduction in sEV particles in CM (Figure 1E). TEM analysis confirmed that size of purified sEV from all states was in the 150 nm range (Figure 1F). Taken together, these observations suggest an altered regulation of MVB biogenesis and/or sEV release during quiescence, which was reversed as cells re-entered proliferation.

### Quiescent myoblast-derived sEVs are enriched for exosome marker proteins

**FIGURE 2:**
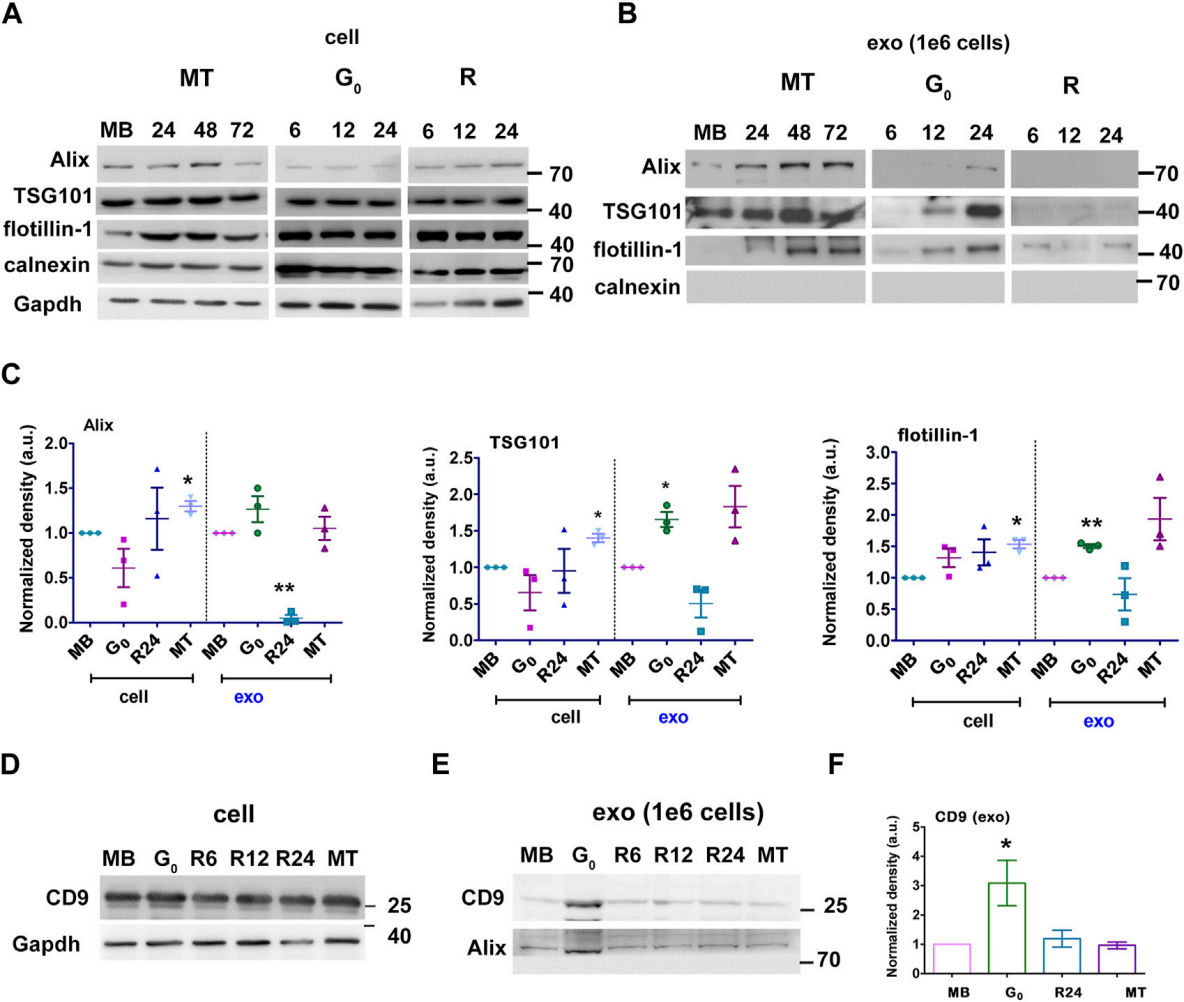
G_0-_sEVs show altered enrichment of exosome marker proteins. Myoblasts were cultured in four different states and exosomes purified at different times for Western blot analysis: proliferating myoblasts (MB); 24, 48 and 72 h after induction of differentiation (MT); 6, 12 and 24 h during entry into G_0_; 6, 12 and 24 h after reactivation from quiescence (R). Abundance of exosomal markers Alix, TSG 101, flotillin and CD9 in total cell-associated and purified sEV fractions was determined to compare enrichment. Controls included non-endosomal ER marker calnexin and loading control GAPDH. **(A)**. Total cell-associated protein (20 µg) isolated from MB, G_0_, R and MT states probed for Alix, TSG101, Flotillin. **(B)**. Protein from sEV fractions normalized to live cell number **(**1 × 10^6^ cells). For G_0_ and R time points, CM was collected for 6 h at 0-6, 6-12, 18–24 h, represented as 6, 12 and 24 h respectively; for MT, CM was collected for 6 h from 18-24, 42-48 and 66–72 h after differentiation and represented as 24, 48 and 72 h respectively; exosomes were purified and probed for Alix, TSG101, Flotillin. **(C)**. Densitometric analysis for Western blots in A and B for Alix, TSG101, Flotillin: for each protein, cell-associated (“cell”) and secreted sEV fractions (“exo”) are shown in separate panels, and normalized against MB-derived sEV. **(D–F)**. CD9 protein expression was probed in Western blots using **(D)** cell-associated lysate from 1 × 10^6^ cells, **(E)** sEV fractions normalized to live cell number **(**1 × 10^6^ cells) and **(F)**. Densitometry analysis of exo CD9. n = 3, significance against MB as determined by paired Student’s t-test, *p ≤ 0.05 and **p ≤ 0.005.

Given the significant increase in sEV output in G_0_, we further characterized these vesicles. We confirmed that the fraction purified by ultracentrifugation was enriched for exosomes using known markers: ESCRT-dependent early endosome proteins Alix and TSG101, lipid-raft protein flotillin, and tetraspanin CD9. Induction of quiescence for 6, 12 and 24 h did not alter the levels of cell-associated Alix, TSG101 and flotillin significantly (Figure 2A cell). However, G_0_ cells (at 24 h) showed significant enrichment in exosome markers compared to MB, consistent with increased vesicle secretion (Figure 2B exo). Cell-associated levels of these markers did not vary much with cell state transitions, while sEV fractions showed variations consistent with changes in overall secretion rate and relative enrichment (Figures 2C–E). Compared to the cell lysate (Figure 2D), expression of tetraspanin CD9 was significantly increased in secreted G_0_ sEV (Figures 2E,F) The sEV fractions from all states were negative for ER marker calnexin, confirming the exosomal enrichment. Taken together, the results indicate that quiescent cells show increased secretion of sEVs and enhanced exosome marker expression.

To confirm our results, we used sucrose density gradient centrifugation to separate and enrich secreted vesicles (Colombo et al., 2014) (Supplementary Figure S3C). TEM of pooled sucrose gradient fractions enriched for the sEV markers confirmed the enrichment of vesicles in the size range (∼150 nm diameter) expected for exosomes (Supplementary Figure S3D). The distribution of markers Alix and TSG101 in different fractions obtained from G_0_, MB and MT were analyzed (Supplementary Figure S3E). For G_0_, exosome markers were enriched in density fractions between 1.12 and 1.14 g/mL, while for MB these markers were enriched between 1.16 and 1.19 g/mL and for MT between 1.10 and 1.16 g/mL. These observations are consistent with differences in exosome cargo density and composition from different cellular states. Absence of calnexin confirmed absence of ER-derived vesicles.

Taken together, we provide evidence that quiescent myoblasts in culture secrete sEVs with exosome properties in higher quantities than proliferating or differentiated cells, and that cell cycle reactivation reverses this increased secretion, suggesting that exosome biogenesis/secretion represents a regulatory node during reversible cell cycle exit.

### Altered lipid accumulation in G_0_: increased lipid droplets and ceramide-rich vesicles

To explore the possible mechanisms behind increased sEV secretion in G_0_, we investigated lipid droplets (LD), which provide a high degree of metabolic flexibility in skeletal muscle (Tan et al., 2021). The status of LD/lipid intermediates in G_0_ myoblasts has not been reported. LD detected by Oil Red O (ORO) (Supplementary Figure S4A) were measured by quantitative imaging (Supplementary Figure S4B). MT showed 2.3-fold higher accumulation of LD as reported (Tan et al., 2021). Notably, we found that G_0_ cells showed ∼3-fold more LD than MB, consistent with altered lipid metabolism in quiescence. Further, the distribution of ceramide-rich membranes (includes MVBs, ER and Golgi) using BODIPY^TR^ ceramide staining (Carayon et al., 2011; Horbay et al., 2022), showed profound architectural changes in G_0_ compared to MB (Supplementary Figure S4C): high-resolution Airy scanning of a single confocal slice revealed numerous vesicles distributed to the periphery in G_0_, whereas scattered fibrillar staining dominated in MB. Conceivably, alterations in LD and ceramide-rich intracellular membranes in G_0_ may be involved not only in fatty acid oxidation which sustains survival in the low metabolic state of quiescence, but potentially for increased sEV biogenesis.

### Increased expression of exosome regulatory proteins including Kibra, Rab27a and tetraspanins in quiescence

**FIGURE 3:**
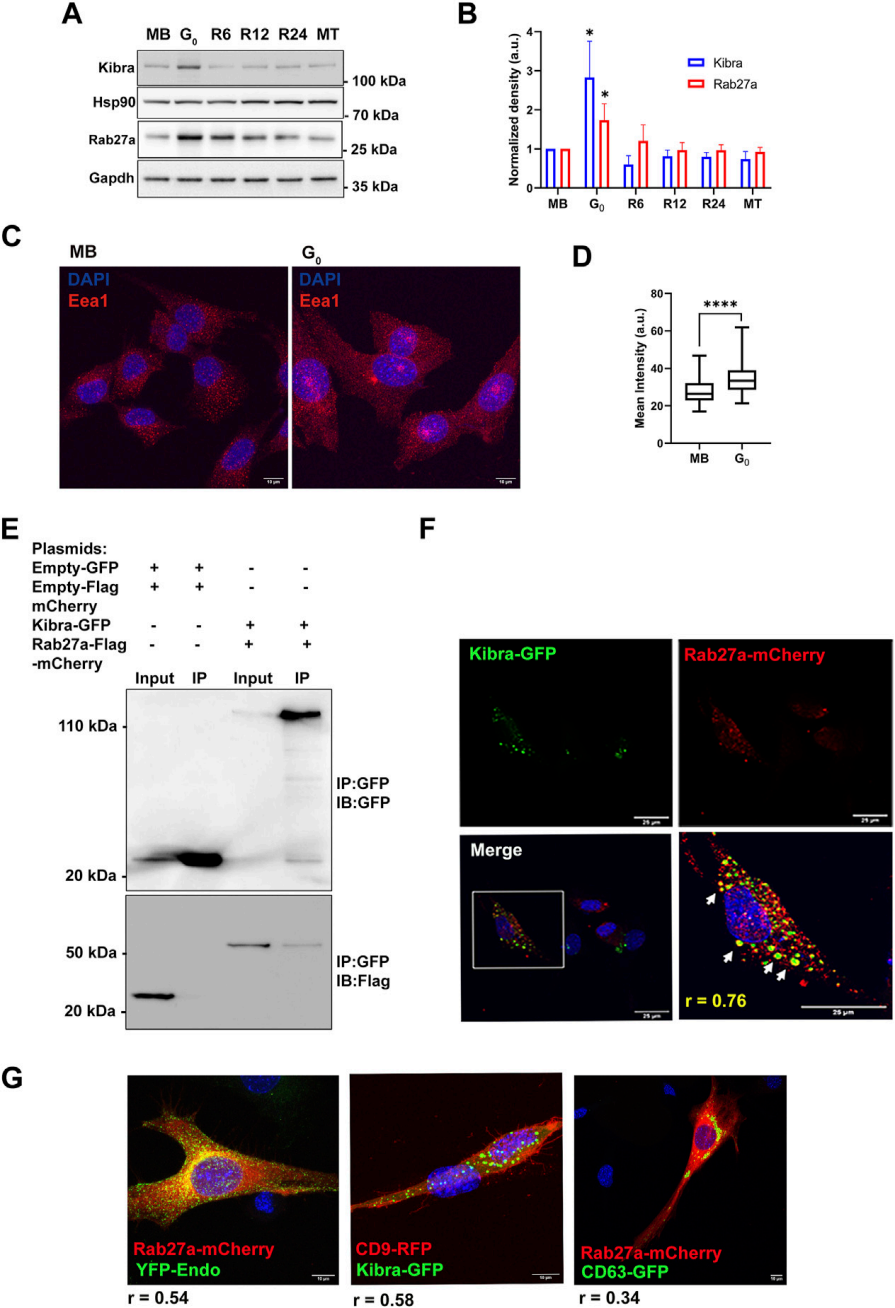
Increased expression of exosome regulatory proteins Kibra and Rab27a in G_0_. **(A)**. Immunoblotting of Kibra and Rab27a protein expression in total cell-associated protein from different cellular states, MB, G_0_, R6, R12, R24, and MT. **(B)**. Quantitation of Western blots shows that expression of both Kibra and Rab27a is enhanced in G_0_. **(C)**. Representative confocal image of Early endosomal antigen 1 (Eea1, early endosome resident protein) showing more abundant endosomal vesicles in G_0_ than in MB. **(D)**. Quantification of mean fluorescence intensity of Eea1 in MB and G_0_. **(E)**. Interaction of GFP-KIBRA and Flag-mCherry-Rab27a: immunoprecipitation using with GFP-trap and blotted for either GFP (upper panel) or Flag (lower panel) (representative IP from n = 3). **(F)**. Confocal microscopic analysis of ectopic GFP-Kibra (green) and endogenous Rab27a (red) in myoblasts showing partial co-localization of Kibra and Rab27a indicated by arrows in the zoomed image; Pearson’s correlation coefficient of colocalization *r* = 0.76; nuclei are stained with DAPI (blue). Scale bar = 25 μm. **(G)**. Co-localization of Kibra/Rab27a with exosome markers in myogenic cells. Representative confocal images of pairs of marker proteins (Rab27a-mCherry:YFP-Endo; Kibra-GFP::CD9RFP; Rab27a-mCherry:CD63GFP). Extent of colocalization as estimated from Pearson’s correlation coefficient (*r*) is indicated in the merged images.

The scaffold protein Kibra has been implicated in regulating MVB biogenesis in neuronal cells (Song et al., 2019; Han et al., 2022). To investigate whether Kibra participates in regulation of exosome secretion in skeletal muscle cells, we measured endogenous (cell-associated) Kibra protein. We found that G_0_ cells expressed significantly more Kibra protein than MB (Figures 3A,B), which was reversed as cells re-entered the cell cycle during reactivation, correlating with exosome secretion.

In neurons, Kibra functions by interacting with Rab27a, a key regulator of exosome biogenesis during ILV formation and MVB docking to PM (Song et al., 2019). G_0_ cells expressed significantly more Rab27a protein than MB (Figures 3A,B), which like Kibra, was reversed during reactivation, and correlated with alterations in exosome secretion. Notably, the early endosome marker Eea1 also showed enhanced expression in G_0_ (Figures 3C,D), consistent with increased MVB-exosome biogenesis during quiescence, since MVBs are of endosomal origin.

To assess whether Kibra and Rab27a proteins interact in myogenic cells as in neurons, we over-expressed both GFP-Kibra and Flag-mCherry-Rab27a in MB. Kibra was pulled down using anti-GFP nanobodies and its presence in GFP-Kibra:Flag-mCherryRab27a protein complex (124 kD, top panel) confirmed by immuno-blotting with anti-GFP (Figure 3E). Further, probing with Flag antibodies confirmed the enrichment of Flag-Rab27 (54 kD, lower panel, Figure 3E) confirming interaction of Kibra-GFP and Rab27a-flag-mCherry. Immuno-localization showed that Rab27a-mCherry co-localized with Kibra-GFP (Pearson’s correlation coefficient 0.76) (Figure 3F). Further, bonafide exosome markers CD63-GFP, CD9-GFP and YFP-Endo (YFP-tagged RhoB GTPase that marks endosome/MVB compartment), all showed considerable colocalization with Kibra/Rab27a in myoblasts (Figure 3G). Taken together, the results indicate a generalized increase in MVB biogenesis in G_0_.

### Kibra regulates enhanced secretion of exosomes in G_0_ cells

**FIGURE 4:**
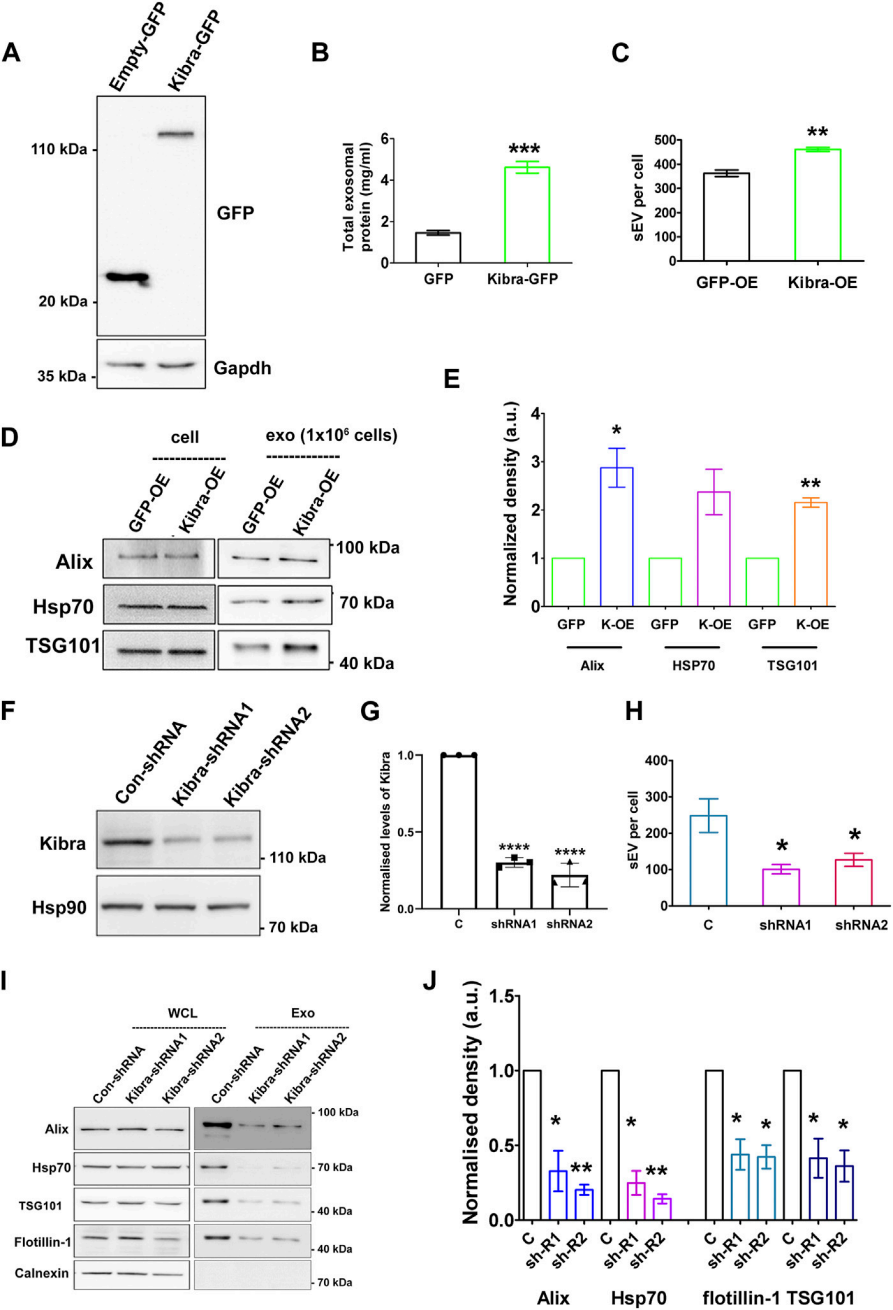
*Kibra positively regulates secretion of sEV in muscle cells* **(A)**. Over-expression of Kibra-GFP in proliferating MB detected by immunoblotting with anti-GFP. **(B)**. Increased protein in sEV fraction collected from equal numbers of myoblasts over-expressing Kibra-GFP or control GFP alone (n = 3). **(C)**. Increased secretion of sEV particles/mL in Kibra-OE condition (NTA analysis). **(D)**. Western blot analysis of secreted sEV harvested from equal numbers of Kibra-expressing (Kibra-OE) or GFP expressing (GFP-OE) cells. Total cell lysate [cell, (1 × 10^6^)] and exosome fraction [Exo, (1 × 10^6^)] were blotted for exosome markers Alix and Tsg101, with HSP70 as a loading control **(E)**. Quantification of sEV proteins obtained from Kibra-OE normalized against GFP-OE cells (n = 3). **(F)**. Decreased total Kibra protein in cell lysates collected from equal numbers of myoblasts expressing shRNA (1 and2) for Kibra (n = 3). **(G).** Quantification of data in **(F)**. **(H)**. Decreased secretion of sEV particles from equal numbers of cells expressing Kibra-shRNA1 and two compared to con-shRNA (NTA analysis). **(I)**. Immunoblot analysis of total cell protein vs. sEV fraction from equal numbers of cells expressing con-shRNA and Kibra-shRNA one and 2. **(J)**. Quantification of exosome markers in sEV fraction from Kibra-shRNA1 and 2 cells normalized against con-shRNA cells (n = 3). All quantification results show the mean ± SE, *p ≤ 0.05, **p ≤ 0.005, ***p ≤ 0.0005 as determined by Student’s *t-*test.

To determine whether Kibra affects sEV secretion rate, we over-expressed GFP-tagged Kibra in MB (Figure 4A). Ectopic expression of GFP-Kibra significantly increased the amount of protein in the exosome fraction by 3-fold over GFP control (Figure 4B), and NTA confirmed 30% increase in exosome numbers in Kibra-overexpressing MB (Figure 4C; Supplementary Figure S5A), along with enhanced expression of exosome markers Alix, Tsg101 and Hsp70 (∼3, 2.5 and 2-fold respectively, Figures 4D,E) in the purified sEV fraction. Thus, Kibra appears to enhance sEV secretion in muscle cells.

To further confirm the involvement of Kibra in sEV release, we inhibited endogenous Kibra expression in MB, using two distinct shRNAs (Figures 4F,G). Suppression of Kibra expression reduced sEV secretion to ∼40% as analysed by NTA, compared to cells expressing a control non-targeting sequence (Figure 4H; Supplementary Figure S5B), and decreased enrichment of exosome markers in the sEV fraction (Figures 4I,J), consistent with a role for Kibra in exosome release. Consistent with the reported role of Kibra in neurons, our results indicate a causal link between Kibra and sEV secretion in skeletal muscle cells as well, where increased expression of Kibra in G_0_ cells is likely to facilitate the associated increase in sEV secretion.

### Proteomic analysis reveals distinct state-dependent sEV protein profiles

**FIGURE 5:**
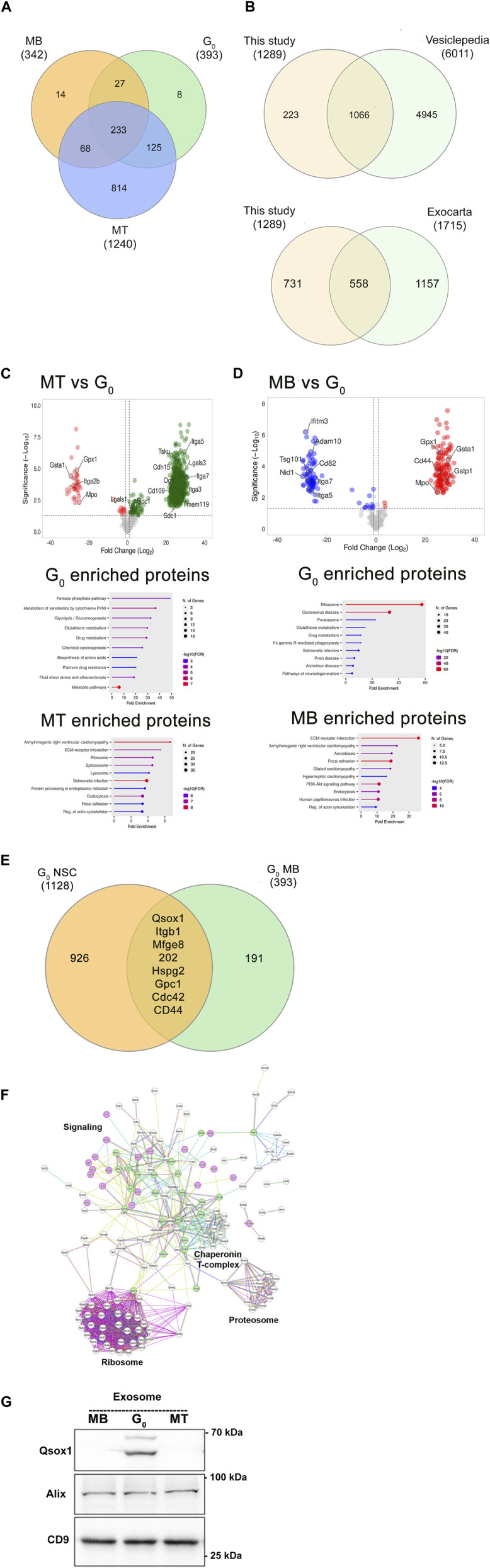
Proteomic analysis of exosomes from different muscle cell states. **(A)**. Comparison of LC-MS/MS analysis of enriched exosome proteins identified in three different cellular states reveals common and stage-specific proteins. **(B)**. Significant overlap between total exosome proteins identified in this study vs Vesiclepedia and Exocarta databases. **(C)**. Volcano plots and gene ontology analysis of differentially enriched exosomal proteins in MT vs G_0_. **(D)**. Volcano plots and gene ontology analysis of differentially enriched exosomal proteins in MB vs G_0_. Proteins of interest are highlighted in volcano plots. **(E)**. A quiescence-associated exosomal enrichment signature: exosome proteins commonly identified from quiescent neural stem cells (qNSC) (Zhang et al., 2021), and quiescent myoblasts (qMB) (this study). **(F)**. Direct protein-protein interaction (PPI) network of quiescence-associated exosome components in qMB and qNSC using String analysis. 198/202 common proteins identified in Figure 5E show direct PPI. Nodes represent proteins (names are indicated), edges represent interactions: known interactions from curated databases or experimentally determined in the literature are colored teal and pink respectively. Other colors represent predicted interactions. Proteostatic machinery complexes (ribosome, proteosome, chaperonins) are noted. Selected protein nodes (circles) have been colored to indicate intracellular signaling proteins (green) or transmembrane receptors, ECM components or secreted proteins (pink). **(G)**. Immunoblot detection of Qsox1 protein in exosomes shows increased enrichment specifically in G_0_, validating the proteomic analysis.

To evaluate whether sEVs released by different cell states showed altered protein profiles we used label-free quantification of LC-MS/MS analysis (Figure 5). A total of 1,289 proteins were reproducibly detected in purified sEVs, with 233 proteins common to three states (MB, G_0_, MT) (Figure 5A). Comparison of the 1,289 collated muscle cell sEV proteins with Exocarta (Keerthikumar et al., 2016) and Vesiclepedia (Pathan et al., 2019) databases (Figure 5B) revealed that >40% of known exosomal proteins including canonical exosomal markers TSG101, CD63, CD9, and CD81 and Alix were enriched, consistent with reproducible isolation of the exosome subclass of sEVs. Notably, the Wnt pathway effector β-catenin (MacDonald et al., 2009) was also found in sEVs from all three states, but there were quantitative differences in β-cat abundance (Mt>G_0_>Mb), and MT sEV also carried Wnt10b and Wnt co-receptors Lrp1 and 4.

A comparison with exosome proteins reported from proliferating and differentiated muscle cells (Forterre et al., 2014) revealed substantial overlap (including Haspa8, Ncam, CD97, Cdh13, Col6a1, Col6a2, Tollip, Lgsals3, and Lgals3bp). As our study also included quiescent myoblast-derived exosomes which have not been previously reported, we looked for proteins selectively enriched in G_0_ compared to MT (Figure 5C) and in G_0_ compared to MB (Figure 5D). The volcano plots and gene ontology analysis highlight the proteins and pathways enriched in these binary comparisons, which together revealed that a signature of glutathione redox-related proteins (GSTA1, GSTP1, Gpx1, and Mpo) was found to be enriched in G_0_-derived exosomes.

To determine if quiescence *per se* is associated with a particular protein signature, we compared proteomic data from quiescent neural stem cell (qNSC)-derived exosomes (Zhang et al., 2021): despite differences due to cell type, 202 proteins were commonly enriched between qNSC and quiescent (G_0_) myoblast-derived exosome profiles (Figure 5E). String network analysis (https://string-db.org, version 12.0) revealed that 198/202 of these quiescence-enriched exosomal proteins showed direct protein-protein interactions (Figure 5F). In addition to the enrichment of ribosomal and proteosomal subunits and the chaperonin T-complex, of particular interest are transmembrane signaling proteins (integrins, CD44-Hyalunoran receptor, Mannose phosphate receptor), ECM components (Laminin, Fibronectin, HSPGs), intracellular signaling components (Cdc42, Rhog, and Rabs), and signaling enzymes (quiescin Qsox1 sulfhydryl transferase, PP2A, MAPK1). Immunoblotting of Qsox1 validated the differential enrichment in G_0_ revealed by the proteomic analysis (Figure 5G). Thus, the quiescent state is associated with enrichment of proteins consistent with altered cargo loaded, suggesting possible differences in uptake and/or signaling capability of exosomes derived from different cell states. Overall, the proteomic analysis confirms that muscle cell derived sEV profiles substantially overlap with those of *bona fide* exosomes, that different muscle cell states are marked by both qualitative and quantitative changes in sEV profiles, and that sEVs from quiescent myoblasts share a common signature with quiescent NSCs.

Taken together, four features characterise the sEV fraction analysed thus far: (i) particle size and density (ii) exosome marker proteomic profile (iii) sensitivity to GW4869 a known inhibitor of exosome biogenesis (iv) sensitivity to perturbation of Kibra, a known regulator of exosome biogenesis. Therefore, we conclude that the sEV fraction under analysis is enriched for exosomes, and henceforth refer to these sEVs as exosomes.

### Exosomes derived from different muscle cell states show differential wnt signaling capacity

**FIGURE 6:**
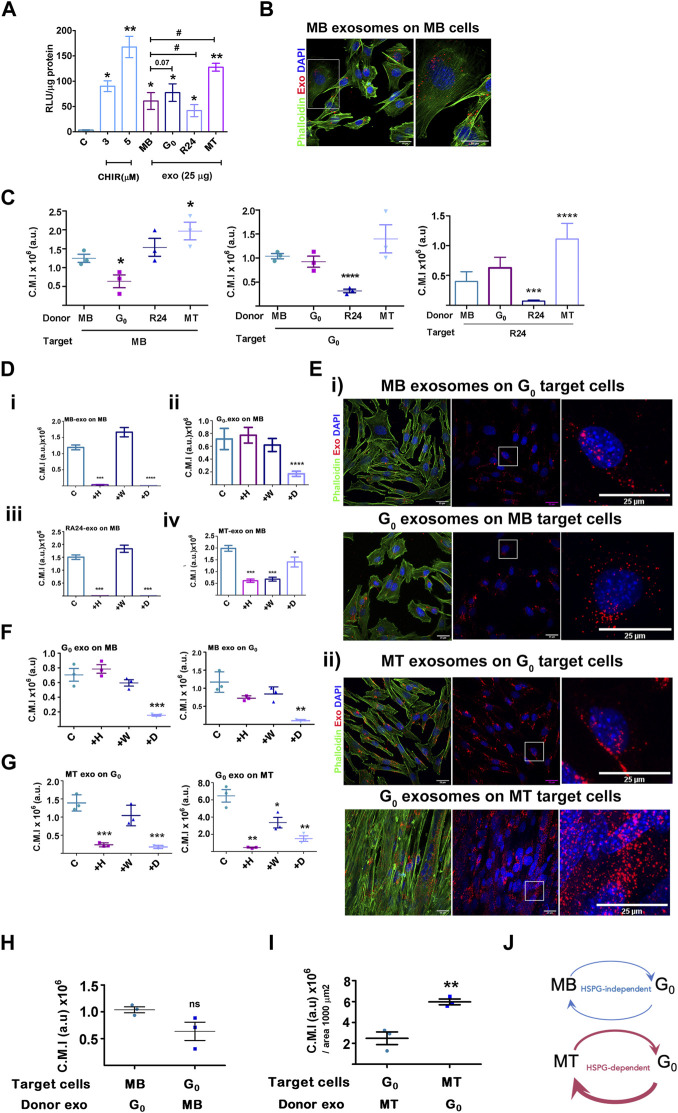
Activation of Wnt signaling and exosome uptake vary with donor and target cell state. **(A)**. Activation of a Wnt transcriptional reporter in target cells by exosomes from different donor cell states. Wnt-TCF transcriptional activity was measured in target MB with stably integrated Tcf/Lef-luciferase reporter TOPflash (canonical Tcf/Lef binding site) or control FOPflash (mutant Tcf/Lef binding site) and treated with Wnt activator CHIR (3 or 5 μM) or 25 μg/mL exosomes derived from different donor cell states as indicated. Tcf/Lef reporter activity is shown as a ratio of TOP to FOPflash luciferase activity normalized to total protein in each sample (RLU/μg protein). Graph represents three independent experiments. Error bars represent mean ± SEM. Significance was determined by Student’s t*-*test. *p < 0.05, **p < 0.005, against control (no additives) and #*p* < 0.05, against MB. **(B–I)** Exosome uptake studies. Purified PKH26-labelled sEV derived from MB, G_0_, R24 or MT donor cells were added to target cells in different states for 4 h, internalization was visualized by confocal microscopy and corrected mean intensity (a.u.) of internalized PKH26-labelled donor sEV after uptake by target cells was quantified. **(B)**. Representative confocal image showing uptake of labelled MB sEV by proliferating MB target cells. **(C)** Internalization of donor exosomes derived from MB, G_0_, R24 and MT by target cells maintained in different cell states**:** Left panel - Target cells: MB; Middle panel - Target cells: G_0_; Right panel- Target cells: Reactivated MB (R24). Quantification is based on measurements of corrected mean intensity of sEV fluorescence (PKH26) internalized by target cells (cell boundaries are visualized by Alexa 488-phalloidin staining of F-actin). 100 or more cells from randomly selected fields in three independent experiments were evaluated. Significance determined by Student’s unpaired *t*-test, *p ≤ 0.05, **p ≤ 0.005, ***p ≤ 0.0005 and ****p ≤ 0.00005. In each graph, comparison is made to MB. **(D–G)** Response to inhibitors of exosome uptake. Target cells were pre-treated for 30 min without inhibitors (control), or with 20 μg/mL heparin **(H)**, 0.5 µM wortmannin (W) or 10 µM Dynole **(D)** followed by co-incubation with PKH26-labelled (red) exosomes (25 µg) for 4 h at 37°C, washed, fixed with 4% PFA, counter-stained with Phalloidin (F-actin, green) and DAPI (nuclei, blue), as in **(B)** and quantitative confocal microscopy of target cells performed. For all graphs, no inhibitor (con), 20 μg/mL heparin (+H), 0.5 µM wortmannin (+W) or 10 µM Dynole (+D). **(D)** Exosome uptake in MB target cells exposed to (i) MB donor exo (ii) G_0_ donor exo (iii) R24 donor exo (iv) MT-donor exo. **(E)** Confocal images of exosome uptake between (i) G_0_-MB pair (ii) G_0_-MT pair. Scale bar: 25 µm. Boxed regions represent zoomed images shown on right. **(F)** Quantification of imaging of G_0_-MB pair after inhibitor treatment. **(G)** Quantification of imaging of G_0_-MT pair after inhibitor treatment. Quantification of uptake is represented as corrected mean intensity (a.u.), based on measurements of at least 100 cells from randomly selected fields derived from three independent experiments, significance determined by Student’s paired *t*-test, ** indicates *p* ≤ 0.005 and *** indicates *p* ≤ 0.0005, in comparison to respective untreated control. **(H)** Relative uptake of exosomes between G_0_-MB pair is of comparable range. **(I)** Relative uptake of G_0_ exosomes by MT is three fold higher than the uptake of MT exosomes by G_0_. **(J)** Schematic representing data in **(C–G)** showing variations in uptake of donor exosomes by different target cell states, as well as differential inhibitor sensitivity. The thickness of the arrows represents the relative uptake. Further, G_0_ exosome uptake by MB is HSPG-independent (heparin-insenstive), but by MT is HSPG-dependent (heparin-sensitive). MT show quantitatively highest uptake of G_0_ exosomes compared to all other conditions.

Given the detection of Wnt pathway components in the proteomic analysis, we tested the functional capacity of muscle cell-derived exosomes to activate Wnt signaling in target cells. Exosomes purified from four different states were tested for their ability to activate an integrated TCF-Lef transcriptional reporter TOPflash in stable Wnt-responsive C2C12 cells (Venugopal et al., 2020). The small molecule Wnt activator, CHIR99201 served as a positive control and was used as an index of activation, inducing TOPflash activity ∼30-fold and ∼55-fold induction at 3 and 5 µM respectively (Figure 6A); the mutant FOPflash showed basal luciferase activity across samples. Equal amounts of exosomes (25 µg) derived from MB, G_0_ and MT induced significant TOPflash activity in the same range as the chemical inducer [MB 20-fold, G_0_ 27-fold, MT 42-fold], whereas R24-derived exosomes were less effective (∼14-fold activation) which can be represented as MT>G_0_>MB > R24. Thus, exosomes derived from different cellular states elicited differential Wnt signaling response, consistent with the proteomics data showing that they differ in their signaling payload (specifically βcat and Wnts).

### Exosomes derived from different muscle cell states show differential uptake by target cells

Exosome-mediated signaling may be affected by factors such as extent and mode of uptake by target cells. We measured the uptake of PKH26-labelled exosomes derived from different donor cell states (MB, G_0_, R24 and MT) by target cells in a single state (proliferating MB). Donor exosomes (25 μg) were added at 37°C for 4 h followed by visualization and quantitatve fluorescence microscopy; cell boundaries and nuclei were visualized by staining with phalloidin and DAPI respectively (Figure 6B). Labelled exosomes from all states were actively taken up by MB (inhibited at 4°C, not shown), but to different extents. G_0_-derived exosomes were least efficiently taken up by MB target cells (Figure 6C), MB and R24-derived exosomes to relatively equivalent levels, and MT-derived exosomes showed the strongest uptake by MB (summarized as MT>G_0_>MB>R24). Notably, the extent of uptake did not correlate with the relative ability of exosomes from these states to activate Wnt signaling (MT>G_0_>MB > R24), suggesting that other parameters such as the route or mechanism of uptake may regulate the final signaling outcome.

### Exosomes derived from different muscle cell states are internalized by distinct pathways

To determine whether exosomes derived from different donor cell states are internalized by different pathways, we used known inhibitors: heparin (competitive inhibitor of cell-surface HSPG-mediated uptake), wortmannin (PI3K inhibitor, disrupts macro-pinosome mediated uptake) and dynole (dynamin inhibitor, disrupts clathrin-mediated endocytic uptake). MB target cells were pre-incubated for 30 min with inhibitors [heparin (20 μg/mL), wortmannin (0.5 µM) or dynole (10 µM)], followed by co-incubation with PKH26-labelled exosomes (25 µg) as described. Consistent with reports that HSPGs participate in exosome uptake, heparin treatment of target cells reduced autocrine/self exosome internalization in all states (Supplementary Figure S6A–C). However, uptake of different donor state-derived exosomes by MB target cells showed differential sensitivity (Figure 6D). G_0_-derived exosomes were internalized predominantly by HSPG-independent, clathrin-dependent pathways, MB and R24-derived donor exosomes were internalized via both HSPG-dependent and clathrin-dependent pathways, and MT-derived exosomes were taken up by HSPG-dependent, clathrin-dependent as well as wortmannin-sensitive pathways. The differential inhibitor sensitivity of uptake by target cells in a single state suggests distinct mechanisms for taking up different donor exosomes. Conceivably, the differential enrichment of integrins, ECM molecules, HSPGs and related proteins in different donor cell states (Figure 5) may play a role. Notably, uptake of G_0_-derived exosomes was HSPG independent.

### Differential uptake between donor-target cell states may suggest directionality of exosome communication

In skeletal muscle, differentiated myofibers far outnumber the G_0_ MuSCs which represent only 1%–3% of nuclei in the tissue and reside in a myofiber niche. Following damage, to regenerate lost tissue, G_0_ MuSCs are activated and give rise to large numbers of proliferating myoblasts. To model the possible communication between these components of muscle using our culture system, we next tested whether exosome uptake between specific donor-target pairs exhibit differential inhibitor sensitivity, using the assay described above.

*MB and G*_*0*_ *donor-target pair not sensitive to HSPG inhibition*: Whereas dynole significantly inhibited the uptake of G_0_-derived exosomes by MB target cells, (Figure 6Dii, Figure 6E), heparin and wortmannin did not. Similarly, when MB-derived exosomes were added to G_0_ target cells (Figures 6E,F), neither heparin nor wortmannin affected uptake significantly, but dynole did. Thus, exosome-dependent cross talk between the donor-target pair of G_0_ and MB-cell states exhibited clathrin-dependent uptake, but is HSPG-independent.

*G*_*0*_ *and MT donor-target pair sensitive to HSPG inhibition*: We similarly assessed inhibitor sensitivity of exosome uptake between G_0_ and MT donor-target pair (Figure 6G). MT-derived exosomes were internalized effectively by G_0_ cells, but in addition to clathrin, uptake was heparin-sensitive. Similarly, internalization of G_0_-derived exosomes by MT was sensitive to heparin. We conclude that internalization of exosomes in G_0_ and MT employs both HSPG-mediated and clathrin-dependent endocytosis pathways, but as distinct from the MB-G_0_ pair, uptake in between MT and G_0_ was HSPG-dependent.

*G*_*0*_ *exosomes are most efficiently internalized by MT*: We assessed relative uptake of G_0_ exosomes by MB vs. G_0_ vs MT, considering uptake as PKH26 fluorescence intensity per unit area. G_0_ exosomes were taken up ∼6-fold less efficiently by proliferating MB (Figure 6H, C.M.I, ∼1 × 10^6^) than differentiated MT (Figure 6I, C.M.I 6 × 10^6^), and at least 3-fold less efficiently than by G_0_ target cells (Figure 6C, C.M.I. ∼0.7 × 10^6^). G_0_ exosomes are enriched for HSPG2 expression (Figure 5), yet internalization of G_0_ exosomes by MT but not by MB is heparin-sensitive (see above), implicating a target cell-dependent mechanism. Since HSPG abundance and glycation diversity are known to increase during muscle differentiation (Ghadiali et al., 2017; Mii and Takada, 2020), we infer that HSPGs on MT may be involved in the higher efficiency of uptake. Overall, the differential sensitivity and extent of uptake of exosomes suggest differences in exosome-mediated crosstalk between muscle cells in different states, with possible directionality of signaling, and differential utilization of HSPG pathways (Figure 6J).

Taken together, the experiments thus far provide evidence of state-dependent heterogeneity of exosomes in terms of quantitiative secretion, proteomic content, extent and pathways of uptake, and Wnt signaling function which is likely an integrated outcome of all of these aspects. In particular, G_0_ exosomes are more efficiently taken up by MT than MB or G_0_, via an HSPG-dependent pathway.

### G_0_-derived exosomes have pro-myogenic effects on target cells: reduced MB proliferation and enhanced differentiation

**FIGURE 7:**
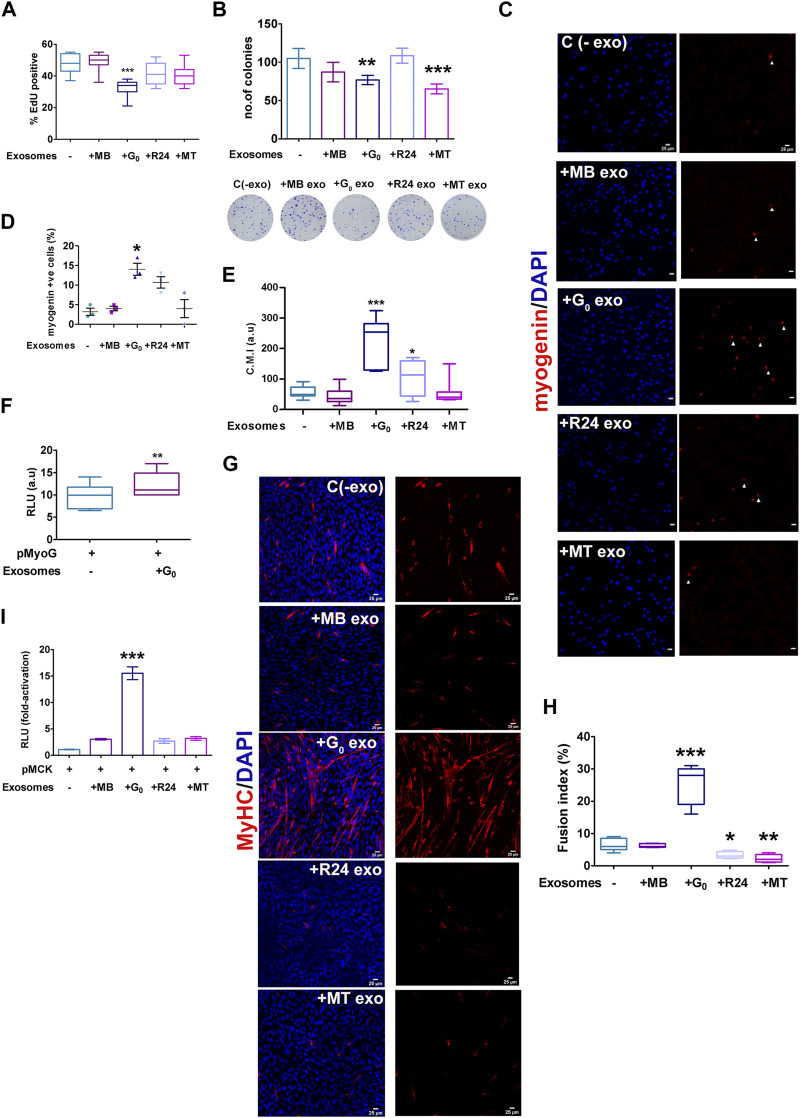
Target cell responses to G_0_ exosomes suggest a novel role in differentiation **(A)** G_0_-derived exosomes suppress proliferation in proliferating myoblasts. Frequency of cells incorporating EdU was measured after exposure of target MB to exosomes derived from different cellular states (MB, G_0_, R24, MT). More than 500 cells were analyzed from three independent experiments. **(B)** Colony formation assay of target MB after exposure to exosomes derived from different cellular states for 24 h, with representative methylene blue stained colonies shown below the graph, *n* = 3. **(C)** Myogenin expression detected by immunofluorescence in target MB treated with exosomes derived from MB, G_0_, R24 and MT donor cells in GM. Images are representative of three independent experiments. Scale bars, 25 µm. **(D)** Frequency of Myogenin^pos^ cells **(E)** Quantification of image intensity of Myogenin expression per cell **(F)** Myogenin promoter-luciferase (pMyoG-Luc) reporter activity following transient transfection and exposure to exosomes for 24 h. Dual-luciferase assay depicts normalized reporter activity. **(G)** Immunofluorescence showing MyHC staining in MB treated with exosomes derived from different target cell states in DM for 24 h. Images are representative of three independent experiments. Scale bars, 25 µm. **(H)** Fusion index of MyHC^pos^ cells. More than 200 nuclei from randomly selected fields of three independent experiments were scored for presence within myotubes of >2 nuclei. **(I)** MCK promoter-(pMCK-Luc) luciferase reporter activity following activity following transient transfection and exposure to exosomes (25 μg/mL) or PBS for 24 h (normalized RLU using dual luciferase assay for each individual condition, and the fold-activation was calculated with respect to the empty vector. Error bars represent mean ± SE, *p < 0.05, **p < 0.005, and ***p < 0.0005.

Given the heterogeneity in cargo and uptake of exosomes derived from different cell states, as well as signaling function with respect to a Wnt reporter, differences in target cell phenotypes may be expected. To determine whether donor exosomes derived from different cellular states elicit distinct phenotypic effects in a single target cell state, target MB were treated for 24 h with donor exosomes from MB, G_0_, R24 or MT, and then assessed for proliferation and differentiation status. While treatment of proliferating MB with exosomes derived from MB, R24 or MT exosomes did not affect EdU incorporation significantly compared to untreated control, G_0_ exosomes suppressed DNA synthesis (Figure 7A). Further, both G_0_ and MT exosomes suppressed colony forming ability (Figure 7B), suggesting that they may carry inhibitors of proliferation and/or self-renewal.

Notably, treatment of proliferating MB with exosomes derived from G_0_ cells induced differentiation, while neither MB nor MT-derived exosomes had this effect. G_0_ exosomes uniquely induced Myogenin expression, as measured by increased frequency of Myogenin-expressing cells (Figures 7C,D), increased intensity of Myogenin expression per cell (Figure 7E), and enhanced Myogenin promoter-luciferase activity (Figure 7F). Further, exposure to G_0_-exosomes led to multinucleated myotube formation with a 4-fold increase in fusion index and strong MyHC expression (Figures 7G,H), accompanied by 14-fold increase in muscle creatine kinase (MCK) promoter-luciferase activity (Figure 7I). Together, these results show that only G_0_ exosomes are pro-myogenic, inducing the entire myogenic cascade, and overt phenotypic fusion into myotubes. Wnt signaling is known to be pro-myogenic (Tanaka et al., 2011; von Maltzahn et al., 2012; Subramaniam et al., 2014; Aloysius et al., 2018). Despite greater enrichment of Wnt signaling components including βcat, Wnt10, Lrp1/4 in MT-derived exosomes, only G_0_-derived exosomes were able to collectively suppress proliferation, reduce self-renewal and induce differentiation in MB target cells. Based on the inhibitor sensitivity profile, G_0_-derived exosomes may interact with target MB via an HSPG-independent mechanism to exert these phenotypic effects. Taken together, our data suggest that integrated signaling (including pathways other than Wnt) triggered by exosome binding/uptake may be responsible for the uniquely promyogenic functions of exosomes from G_0_ cells.

**FIGURE 8:**
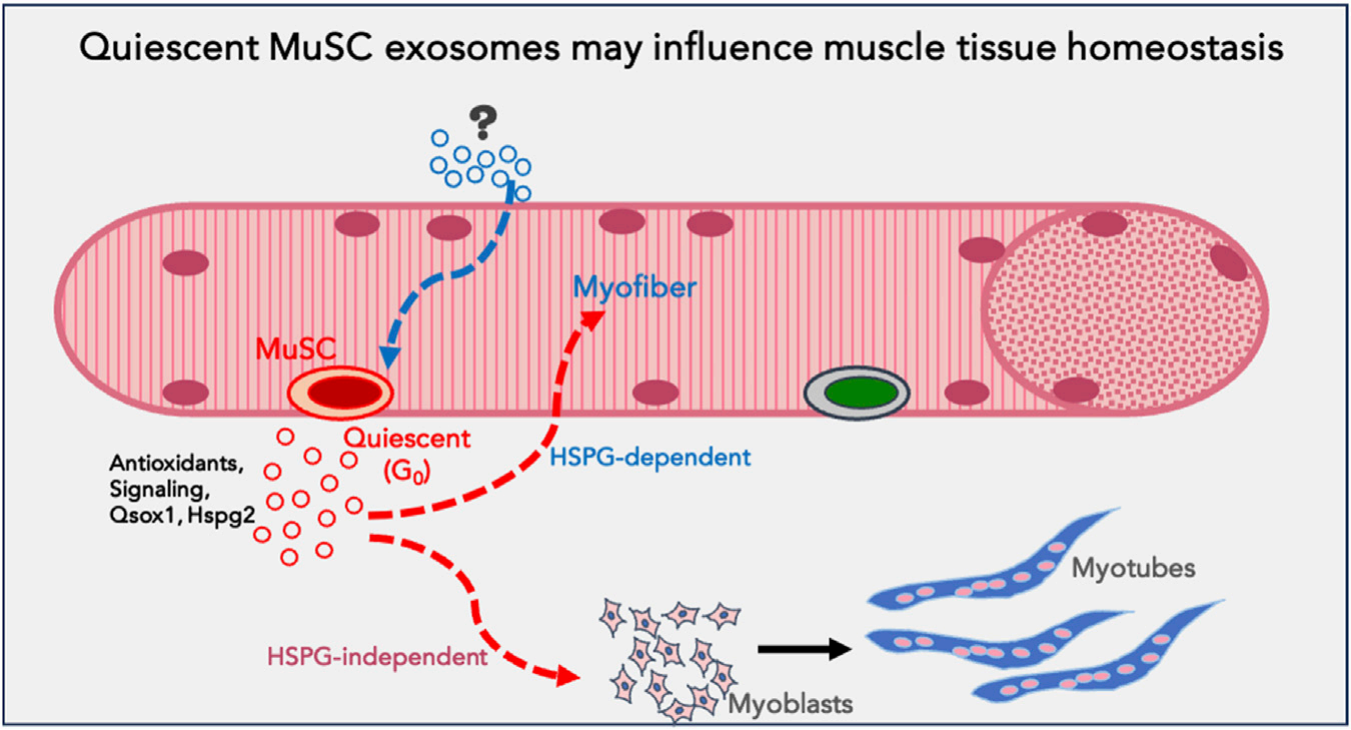
*Model proposing a function for quiescent MuSC exosomes in muscle tissue*: Based on the data obtained using quiescent myoblasts in culture, we propose that quiescent muscle stem cells *in vivo* may be a source of homeostatic signals that support myofiber health or stress-tolerance (antioxidants), reinforce differentiation in uninjured muscle fibers and promote differentiation of proliferating myoblasts during regeneration (signalling). G_0_-MuSC exosomes uptake into myofibers may be via HSPG-dependent mechanisms, but into myoblasts via HSPG-independent mechanisms.

Overall, this study reveals that proliferative and differentiation status of a single celltype influences secretory output as well as differential uptake of exosomes. This heterogeneity suggests the potential for altered signaling crosstalk as cells transition between distinct cellular states. In particular, quiescence in muscle cells is associated with enhanced exosome output and altered exosome cargo; these G_0_-derived exosomes exhibit higher capacity for inducing differentiation in MB and greater uptake by MT. In summary, the results suggest that quiescent muscle cells have a greater capacity for signaling cross talk than previously appreciated, by enhancing differentiation of proliferating myoblasts, and/or by reinforcing resilience/differentiation of myotubes (Figure 8).

## Discussion

In this study, we have investigated the secretion of sEVs from skeletal muscle cells. Using a culture model which allows the recapitulation of distinct physiologically relevant cellular states, we reveal a state-specific regulation of the quantity, protein profile, uptake and function of sEVs. We use multiple criteria to characterize the purified sEVs as exosomes. Unexpectedly, quiescent cells secreted more exosomes than other cell states, and exosomes from quiescent myoblasts are uniquely able to induce differentiation in target cells. Together, these findings suggest the intriguing possibility that despite depressed metabolic activity and low numbers *in vivo*, quiescent cells may play an unexpectedly prominent role in influencing other cells in their environment via their secreted exosomes.

The role of exosomes in skeletal muscle biology is not well understood (Bittel and Jaiswal, 2019). Emerging roles of EVs in different systems have highlighted the challenge posed by the heterogeneity between subclasses of EVs, and emphasize the need to identify cell type- or cell state-specific attributes before assigning biological significance (Aoi and Tanimura, 2021; Hanson et al., 2023). Skeletal muscle tissue is reported to release EVs that regulate both muscle and other organs by delivering specific miRNAs and proteins in normal (Guescini et al., 2010; Su et al., 2018) and disease conditions (De Gasperi et al., 2017; Guescini et al., 2017). In regenerating muscle, activated MuSC secrete exosomes containing miRNAs that prevent excessive collagen biosynthesis by resident fibroblasts (Fry et al., 2017). However, there are no reports on sEVs released by quiescent MuSC.

Quiescent MuSC *in vivo* are rare, mitotically inactive and exhibit repressed transcription, translation and metabolism (Ancel et al., 2021). While a low metabolic state in quiescent MuSC is known to play an adaptive role in their resilience and regenerative ability (Benjamin et al., 2022), the potenial signaling impact of the rare dormant MuSC population on the bulk of tissue in homeostasis has not been explored. During the response to muscle damage, activation of quiescent MuSC leads to their expansion, self-renewal and differentiation, which is vital for restoring lost tissue. While the identity and source of signals that activate resting MuSC has been extensively studied (Luo et al., 2005; Bentzinger et al., 2012; Rodgers et al., 2014; Baghdadi and Tajbakhsh, 2018), the influence of the “inert” quiescent MuSC on other cells in uninjured tissue is not well understood, but physical contacts with their neighbors (Krauss and Kann, 2023) and secreted factors including exosomes are potential mechanisms. Cell type- or state-specific markers of exosomes are not available for muscle, making the direct identification of the source of exosomes *in vivo* a challenge. Therefore, we used muscle cells cultured to achieve different states (quiescent, proliferative, reactivated and differentiated) that represent different stages of muscle formation and regeneration *in vivo*, and studied their exosome profile and signaling characteristics.

By comparing the exosome output of quiescent myoblasts to other muscle cell states *in vitro*, we made several unexpected findings: First, G_0_ myoblasts secrete a significantly higher number of exosomes than their exponentially proliferating counterparts. Notably, exosome secretion reverts to lower levels when G_0_ cells are reactivated, indicating a regulated quiescence-dependent mechanism. Second, Kibra, a Rab27-interacting scaffold protein that controls levels of EV secretion is expressed at higher levels in G_0_. Third, while G_0_ exosomes showed a small number of uniquely enriched proteins compared to other muscle cell states, a common quiescence signature in exosome proteins could be discerned between muscle and neural stem cells. Fourth, uptake studies suggest that G_0-_derived exosomes are taken up at higher rates by MT, using an HSGP-dependent pathway, while uptake of G_0_ exosomes by MB is HSGP-independent. Finally, exosomes from both G_0_ and MT cells have higher Wnt signaling capability than exosomes from proliferating or reactivated MB, but G_0_-derived exosomes are uniquely capable of inducing myogenic differentiation, consistent with their distinct proteomic profile. Together, these results suggest that exosome biogenesis is regulated in quiescent myoblasts at the level of quantity, protein cargo diversity and signaling function. The context and implications of these unexpected findings are discussed below.

Our finding that quiescent MB exhibit enhanced sEV secretion was surprising since entry into a reversibly quiescent state is defined by drastically reduced RNA and protein synthesis and overall metabolism (Ancel et al., 2021; Gala et al., 2022). Lipid metabolism in quiescence varies in different cell types, and is less well understood. Quiescent yeast cells accumulate neutral lipids in G_0_ (Peselj et al., 2022) in a nutrient-dependent manner, and while quiescent mammalian cells such as HSCs (Giger et al., 2020) and T cells (Omar et al., 2017) show high levels of lipid oxidation, quiescent fibroblasts exhibit robust lipid synthesis (Lemons et al., 2010). We found that G_0_ myoblasts show enhanced accumulation of LD and ceramide-rich vesicles, which would in principle support increased sEV formation. Thus, it is possible that the enhanced neutral lipid accumulation serves as a source for increased exosome secretion. The reduction in exosome output by treatment with GW4869 an inhibitor of ceramide synthesis is consistent with this hypothesis. Interestingly, serum or amino acid starvation of fibroblasts has recently been shown to enhance exosome release (Ganig et al., 2021), which taken together with reports that the TSC-mTOR axis controls both exosome biogenesis and autophagy (Zou et al., 2019) may underlie the quiescence-dependent increase in exosome secretion. However, the energy expenditure of enhanced sEV biogenesis/secretion in a metabolically suppressed state is intriguing and remains an open question.

Our results show that increased sEV secretion in G_0_ is modulated by the scaffold protein Kibra, which is known to regulate exosome secretion in neurons (Song et al., 2019) via stabilizing Rab27a, which regulates MVB biogenesis. We confirmed that in muscle cells ectopically expressed Kibra interacts with endogenous Rab27a, and leads to increased exosome secretion, which was reduced when Kibra was knocked down. Taken together with increased Kibra expression in G_0_, these experiments support a role for Kibra in regulating enhanced exosome production and secretion in G_0_. Interestingly, the Kibra-associated protein PatJ has been reported to interact with TSC1 (Singh et al., 2020), suggesting a possible intersection of these two pathways to impact the mTOR controlled balance of autophagic flux and exosome secretion in quiescent cells.

Qualitatively, sEVs secreted by G_0_ cells in culture exhibit morphology and size that are typical for exosomes, as reported earlier for exosomes secreted by MB and MT (Forterre et al., 2014). Proteomic profiling showed that sEVs from all muscle cell states showed enrichment of typical exosome markers CD9, CD63, TSG101, Alix, and flotillin-1. Heterogeneity in exosome function has been reported (Vyas et al., 2014; Willms et al., 2018) but distinctions between different cellular states of a single cell type have not been extensively studied. A recent study using quiescent neural stem cells (qNSC) in culture reported a distinct proteomic profile (Zhang et al., 2021), but did not assess signaling capability. qNSC exosomes were enriched for translation factors and ribosomes, leading the authors to hypothesize that exosome-mediated “discarding” of translation machinery is required for the suppression of protein synthesis typical of G_0_ cells, supported by the finding that inhibition of exosome biogenesis in qNSC led to enhanced protein synthesis. We found that G_0_ myoblast exosomes were also enriched for ribosomal and proteosomal components, consistent with the known global reduction of both protein synthesis and turnover in G_0_ (Hanna et al., 2012; Venugopal et al., 2020). Notably, exosomes from both qNSC (Zhang et al., 2021) and G_0_ myoblasts showed selective enrichment of Qsox1, a secreted sulfhydryl transferase earlier reported from quiescent fibroblast conditioned medium (Ganig et al., 2021), and found to be partially associated with exosomes (Millar-Haskell et al., 2022). By regulating protein sulfation, Qsox1 is thought to modulate ER-resident glycosyl transferases which in turn affect glycosylation of ECM proteins and proteoglycans (Millar-Haskell et al., 2022). Given that exosomes from G_0_ myoblasts were also enriched for proteoglycans such as HSPG2 and glypican-1, as well as several glycan-binding receptors such as the mannose-6 phosphate receptor and the hyaluronan receptor CD44, it is conceivable that altered glycosylation plays a role in cell state-dependent uptake/signaling mediated by exosomes.

The differential uptake or signaling function of exosomes from different cell states has not been previously explored. We found that in muscle cells, both uptake by target cells and consequent signaling differed between cell states, which has implications for cross talk between cells in a tissue. Exosomes can elicit signaling responses in recipient cells by binding and activating receptors at the plasma membrane, and/or by releasing their contents upon internalization. Preferential uptake of exosomes by target cells has been reported between cell types: for example, astrocytes preferentially take up EVs secreted by primary neurons rather than by neuroblastoma cells (Chivet et al., 2014), while HeLa cells do not discriminate (Costa Verdera et al., 2017). In our study, exosomal uptake varied with both donor and target cell states, suggesting the potential for preferential signaling between particular states.

Exosome internalization occurs by different routes (i) by engaging surface proteins via HSPG receptors (Chen and Brigstock, 2016), (ii) by direct internalization via macro/micro-pinocytosis/phagocytosis (Tian et al., 2010), or (iii) by receptor-mediated (clathrin/caveolin dependent) endocytosis (Fitzner et al., 2011). Hspg2 and Glypican are enriched on G_0_ exosomes, but interestingly, heparin-senstive uptake was seen only in particular target cell states. By comparing different donor-recipient pairs of cell states, we determined that rates and routes of uptake can vary even in a single cell type. In the G_0_-MB pair, uptake was relatively similar, and was HSPG-independent. In the G_0_-MT pair, G_0_ exosomes were more efficiently taken up by MT than *vice versa*, and uptake was HSPG-dependent. Also, the extent of uptake of G_0_ exosomes by MT was substantially higher than by MB. Since the G_0_ exosomes are a common factor but the target cells are different (MB vs. MT), the origin of the difference in heparin-sensitivity must depend on the target cell. In this context, the reported increase in cell surface expression and modification of heparan sulfates and HSPGs during myogenesis (Ghadiali et al., 2017), suggests a possible mechanism for preferential heparin-senstive uptake of G_0_ exosomes by MT. Taken together, these state-dependent differences suggest that selective/directional uptake of exosomes may maintain cell state and homeostasis. In particular, the unexpected finding that G_0_-derived exosomes are more abundant than other cell states and preferentially internalized by MT suggests a previously unappreciated signaling role for quiescent cells.

The potential impact of exosomes in target cells was tested using reporter assays as well as endogenous markers as readouts. G_0_ exosomes were uniquely capable of activating myogenic markers in MB target cells: both proliferation and self-renewal were decreased, while expression and promoter activity of the early muscle differentiation regulator Myogenin were enhanced, along with phenotypic differentiation into multinucleated MyHC + myotubes showing enhanced MCK promoter activity. At present, the mechanism for the enhanced promyogenic capacity of G_0_ exosomes is unclear, but given their enriched content of Qsox1 and Hspg2, it is possible that modulation of glycosylation of signaling components such as proteoglycans may play a role. In addition, the enriched antioxidant cargo may support target cell survival or resilience.

Maintenance of cellular homeostasis involves multiple mechanisms including exosomal secretion of factors that may alter cell state. For example, Wnt factors are promyogenic (von Maltzahn et al., 2012), and known to be associated with exosomes (Beckett et al., 2013). Previously, we showed that conditioned media from G_0_ cells contains Wnt (Subramaniam et al., 2014), and the proteomic profile (Figure 5) shows βcat as well as other pathway components in all states, albeit at different levels. MT-derived exosomes are the strongest activators of Wnt reporters, which is in agreement with the finding that adult muscle sustains Wnt activation to maintain differentiation (Tajbakhsh et al., 1998). As MT-exosomes are internalized efficiently by G_0_ cells and *vice versa*, it is conceivable that in muscle tissue, exosome-mediated Wnt signals from quiescent MuSC are received by myofibers for the reciprocal maintenance of their cellular states. In this model, MuSCs may expel Wnt activators/targets in exosomes to suppress precocious differentiation and maintain their quiescent state, while myofibers may benefit from taking up these MuSC-derived exosomes where Wnt activation would reinforce their differentiated state. The observation that while MT exosomes have the strongest Wnt signaling capacity they are not able to induce differentiation in target cells suggests additional mechanisms at play.

At present, the unique ability of G_0_-derived exosomes to induce myogenesis in target cells remains unexplained, but several factors deserve consideration. Since the myogenesis assay was performed with equal amounts of exosomes purified from the four distinct cell states, the inherently higher secretion rate by quiescent myoblasts is not a factor. The extent of uptake of G_0_ exosomes by MB was less than that of other donor exosomes, and is therefore also unlikely to play a role. While it is conceivable that cargo derived from different cell states are differentially degraded after uptake, at present there are no studies that address this issue. The induction of Wnt signaling by G_0_ exosomes was strong, but less than that elicited by MT exosomes, and therefore cannot on its own explain the induction of myogenic differentiation in target cells. Uniquely, uptake of G_0_ exosomes by MB was HSPG-independent, which may provide a clue to their role in myogenic signaling and provides an avenue for future investigation. Finally, the proteomic profile of G_0_ exosomes had some uniquely enriched components (membrane receptors, antioxidant enzymes) which may affect their signaling ability. Thus, our working hypothesis for the unique myogenic function of G_0_ exosomes is that they may carry a differentiation-signaling cargo and that their mode of interaction with target cells delivers these signals via a pro-differentiation pathway.

Our findings using cultured cells have implications for muscle tissue. Quiescent MuSC represent a very minor proportion of cells in total muscle tissue *in vivo* and as such have not been considered a source of signals that influence uninjured muscle. While dormant MuSC must be activated to play a vital role in regenerating damaged muscle, their role in cell-extrinsic signaling in resting muscle has not been sufficiently explored. Our finding that G_0_ myoblasts in culture increase their exosome output and that these G_0_-derived exosomes have the ability to enhance differentiation raises the novel possibility that even dormant cells may participate in tissue homeostasis via exosomes. The quiescence program (Coller et al., 2006; Subramaniam et al., 2014; van Velthoven et al., 2017) that sustains MuSC-intrinsic functions via cell-autonomous mechanisms might simultaneously sustain the surrounding myofibers via antioxidant support, or reinforce their differentiated state via secreted promyogenic signals.

In summary, we report quantitative, qualitative and functional differences in exosomes derived from different myogenic cell states. In particular, quiescent myoblasts (G_0_) secrete higher numbers of exosomes which show more potent pro-myogenic activity than exosomes from other cellular states. Taken together with the enhanced uptake of G_0_ exosomes by differentiated myotubes, our findings have implications for cross-talk between different cellular states of a single cell type, and suggest that resting stem cells may play a signaling or supportive function even in homeostatic conditions, prior to their activation as mediators of repair.

## Materials and methods

### Materials

The following reagents were used; 2, 3-Butanedione monoxime (BDM), (B0753-100G, Sigma), Heparin (Sigma) and Wortmannin (Calbiochem) Dynole (ab120463, Abcam), CHIR99021 (StemRD), Oil Red O (Sigma) 4% Tryphan blue (Invitrogen), Alexa fluor 488 Phalloidin (A12379), BODIPY (D7545, Thermo-Fisher) and DAPI (Invitrogen). Kits: Click-iT EdU Imaging Kit with Alexa Fluor 647 Azide (C10086, Thermo Fisher Scientific), PKH26 Red Fluorescent Cell linker for General Cell Membrane (MIDI26, Sigma-Aldrich), One-Glo Luciferase assay system (Promega). Antibodies for Western blotting were: mouse anti-Alix (ab117600, Abcam), rabbit anti-flotillin 1 (F1180, Sigma), mouse anti-TSG101 (T5701, Sigma), rabbit anti-CD9 (EXOAB-CD9A-1), rabbit anti-Rab27a (17817-1-AP), rabbit anti-Eea1 (2411S), rabbit anti-calnexin (C4731, Sigma), Myogenin (sc12732, Santa Cruz), p130 (SC-317, Santa Cruz), HSP70 (ADI-SPA-820, Enzo), HSP90 (sc-13119, Santa Cruz), Kibra (8,774, CST) and mouse anti-GAPDH (ab8245, Abcam). HRP-conjugated secondary antibodies were from Calbiochem. Details of antibody dilutions for blotting and immunofluorescence are provided in Supplementary Table 2.

### Cell culture

C2C12A2, a strictly anchorage-dependent subclone (Sachidanandan et al., 2002) of mouse C2C12 skeletal muscle myoblasts (Yaffe and Saxel, 1977; Blau et al., 1983) were used in all experiments. All media were supplemented with 1% penicillin and streptomycin (Invitrogen). Only cells that had been maintained in strictly subconfluent conditions were used for preparation of frozen stocks; all experiments were performed on cells between passage two to five after revival from a frozen stock, and monitored for appropriate expression of proliferation and differentiation markers. Adherent proliferating myoblast cultures (MB) were maintained in high serum growth medium [GM; high-glucose Dulbecco’s modified Eagle’s medium (DMEM) with 20% fetal bovine serum; Invitrogen]. For induction of quiescence (G_0_), proliferating MB were treated with 2,3-Butanedione monoxime (BDM 30 mM) in GM for 24 h as described (Dhawan and Helfman, 2004; Venugopal et al., 2020). For reactivation (R) of G_0_ cultures, 24 h after BDM treatment, G_0_ cells were induced to re-enter the cell cycle by removing BDM-media and replacing with fresh GM for different times (6, 12 or 24 h). For induction of differentiation (MT), cultures at 80% confluence were incubated in low serum differentiation medium (DM: DMEM with 2% horse serum), replaced every 24 h for 3 days.

Preparation of Exosome Production Medium (EPM): Exosomes were depleted from 20% FBS or 2% Horse serum-containing medium by centrifugation overnight at 110,000 × *g*, 4°C as described (Thery et al., 2006) and 0.22-µm filter sterilized after adding appropriate supplements for each culture condition. EPM was tested for efficiency of proliferation and differentiation (Supplementary Figure S1F).

### Isolation of sEVs “exosome fraction”

Exosomes were isolated as described previously (Thery et al., 2006). In brief, C2C12 were cultured in different states as described above (MB, G_0_, R6, 12 or 24 h, or MT day 3) and switched to fresh EPM only for collection of exosomes for 6 h (conditioned medium, CM), following which exosomes were isolated by differential centrifugation as follows: 300 × g for 10 min to remove dead cells, 2000×g for 10 min to remove residual debris and apoptotic bodies, 10,000×g for 40 min to remove microvesicles, 0.22 µm filtration to remove vesicles larger than 200 nm and finally ultracentrifugation for 110,000×g for 1.5 h to collect sEV/exosomes. The resulting pellets were resuspended, washed once in double-filtered PBS (DPBS) and re-pelleted at 110,000×g for 1.5 h in a Beckman Coulter Ultracentrifuge (Optima XPN), with Type 45 Ti fixed angle, and 94 mL tubes (Beckman Coulter). The final “exosome” pellet was resuspended in DPBS, and protein concentration measured by a BCA Protein assay kit (Thermo-Fisher). Exosome pellets were resuspended in DPBS and stored in aliquots at −80°C.

### Sucrose density gradient centrifugation

Linear sucrose gradients were prepared using two sucrose solutions corresponding to 60% (w/w) and 5% (w/w) sucrose in 1 × TNE buffer (25 mM Tris–HCl pH 7.5; 0.15 M NaCl; 1 mM EDTA). 6 mL of 60% sucrose solution was placed in the bottom of an ultra-clear ultracentrifuge tube, overlaid with 6 mL of 5% sucrose solution, sealed and allowed to equilibrate overnight at 4°C. 1.5 mL of sucrose solution was carefully removed and 0.5 mL of exosome suspension prepared as above was gently overlaid on the preformed sucrose gradient, and centrifuged to equilibrium (100,000×g for 16–18 h at 4°C using swinging bucket rotor, SW41Ti rotor, Beckman Coulter). 15 fractions of 750 μL each were carefully collected from the top of the tube. 10 μL of each fraction was used to measure the refractive index (Brixxus-CRI375P, Sartorius) and density was determined using the rotor specification for Beckman Coulter. The fractions were individually washed by centrifugation in double-filtered chilled PBS buffer and the final pellet solubilized with 2x Laemmli sample buffer. Individual fractions were displayed using SDS-PAGE, transferred to PVDF and the blots probed as below.

## SDS-PAGE and immunoblotting

Freshly isolated exosome pellets and total cell pellets were resuspended in 2X laemmli buffer. Proteins were resolved by SDS-polyacrylamide gel electrophoresis and transferred to PVDF membranes (Bio-Rad): For detection of exosome markers in cell-associated protein, equal amount of total cell protein (WCL; 20 µg) was loaded; For detection of exosome markers in purified exosome fractions, exosomal protein derived from 1 × 10^6^ secreting cells were loaded. Blots were blocked with 5% milk in TBS containing 0.1% Tween 20, incubated with primary antibodies (antibodies against exosomal markers (Alix, TSG101, flotillin-1, CD9) and an ER marker, calnexin) at 4°C overnight, washed in TBST, and incubated with HRP -conjugated goat anti-mouse or anti-rabbit IgG for 45 min at room temperature. After washing in TBST, the blots were developed using chemiluminescence solutions and imaged using Image Quant. Results were analysed with ImageJ software (NIH, Bethesda, United States).

### Nanoparticle tracking analysis

Exosome size distribution and particle number was determined by nanoparticle tracking analysis (NTA) using a NanoSight system NS300 instrument equipped with a 488 nm laser module (Malvern, United Kingdom) and NTA 3.2 analytical software. The NS300 uses Brownian motion of nanoparticles dispersed within a liquid as its principle of operation. With the aid of a magnification microscope, the laser beam traverses the sample chamber identifying the nanoparticles. Each experiment was carried out in triplicate. Exosome samples obtained from equal number of secreting cells over a 6-h collection period were diluted in DPBS (100-fold) appropriately and injected into the sample chamber. The samples were mixed initially to prevent clumping of nanoparticles which is known to affect the scattering patterns. The experimental setup for NTA was performed on the settings of camera level: 12-13, capture duration: 30 s, no. Of scans: 3 with detection threshold of: 3-4. An average of three scans were taken to represent the particle size and number. The videos were processed by the NTA software, version (NanoSight NTA 3.2) and processed for analysis. Each video yielded the mean, mode, and concentration of particles of the diluted sample (1 mL). The generated data were used for statistical analysis.

### Electron microscopy

Exosomes were evaluated morphologically through negative staining (Thery et al., 2006). The exosome pellet derived from equal number of secreting cells were fixed by resuspension in 100 µL of 2% PFA, 5 µL was deposited on Formvar-carbon coated EM grids (Electron Microscopy Sciences) and allowed to adsorb for 20 min in a dry environment. Grids were washed in 100 µL PBS, fixed in 50 µL of 1% glutaraldehyde for 5 min, followed by seven washes in 100 µL changes of distilled water. The grids were placed in a 50 µL drop of 2% uranyl-oxalate solution pH seven for 5 min followed by contrasting and embedding in a mixture of 4% uranyl acetate and 2% methyl cellulose (1:10) for 10 min on ice in dark. Excess fluid was blotted to leave a thin film over the exosome side of the grid, air-dried and observed under the electron microscope (JOEL JEM 2100) at 120 kV and images captured using Gatan Ultrascan CCD camera. Samples were imaged at ×6700 magnification (scale bar 500 nm).

### LC-MS/MS analysis

Exosome pellets isolated by ultracentrifugation as above and lysed in SDS Lysis buffer (0.2 M Tris–HCl pH 6.8, 8% SDS, 0.05 M EDTA, 4% 2-mercaptoethanol, 40% glycerol, 0.8% bromophenol blue). 50 μg of each exosome lysate were separated on a 15% SDS-PAGE gel, proteins visualized by staining with Coomassie brilliant blue R250. Each lane was individually sliced into 10 pieces, and each piece individually cut into smaller pieces (1–2 mm) for processing. Proteins were subjected to reduction, alkylation, and in-gel digestion using Trypsin (Shevchenko et al., 1996). Digested peptides were desalted and enriched using Pierce C18 Tips. Eluted peptides were resuspended in 5% (v/v) formic acid and sonicated for 5 min (Sennels et al., 2009). Samples were analyzed on Orbitrap Exploris™ 240 mass spectrometer (Thermo Scientific) coupled to a nanoflow LC system (Easy nLC II, Thermo Scientific). Peptide fractions were loaded onto a PepMap™ RSLC C18 nanocapillary reverse phase HPLC column (75 μm × 25 cm) and separated using a 60 min linear gradient of the organic mobile phase [5% Acetonitrile (ACN) containing 0.1% formic acid and 95% ACN containing 0.1% formic acid]. For identification of peptides the raw data was analyzed on MaxQuant proteomics computational platform (Ver. 1.6.8) (Cox and Mann, 2008) and searched against UniProt amino acid sequence database of *Mus musculus* (release 2022.04 with 17,131 entries) and a database of known contaminants. MaxQuant LFQ (Label Free Quantification) feature was used to quantify the differences in abundance between the different exosome states. Proteins with peptide count two or higher were selected for further analysis.

## Data availability

The MS-based proteomics data of all these experiments have been deposited to the ProteomeXchange Consortium via the PRIDE partner repository (Suthar and Ashish, 2014) with the dataset identifier PXD045797.

Gene ontology analysis was performed using ShinyGO 0.77 (http://bioinformatics.sdstate.edu/go/). The String database (https://string-db.org/) (Szklarczyk et al., 2015) version 12.0 was used to analyse protein networks in the 203 common proteins identified between quiescent NSC (Zhang et al., 2021) and this study. The network settings used were 0.400 confidence (medium), and dis-connected nodes were removed: 198/202 proteins fulfilled the criteria. In a String network, nodes represent proteins and edges represent shared physical complexes. Known interactions are from curated databases or experimentally determined in the literature. Other interactions are predicted by the String algorithm from co-association analysis.

### Immunoprecipitation (IP)

Immunoprecipitation of Kibra-gfp was performed according to ChromoTek GFP Trap agarose (gta-20) manufacturer’s protocol. Transfected cells were washed twice with ice-cold PBS and lysed with lysis buffer (10 mM Tris/Cl pH 7.5, 150 mM NaCl, 0.5% NP40, and 0.5 mM EDTA) containing 1X protease and phosphatase inhibitors. 200 μL of lysis buffer was added to 60 mm culture plate and cells were allowed to stand in the lysis buffer for 2 min on ice, after which, cells were scraped and collected in a 1.5 mL microcentrifuge tube. Lysate was sheared through a 2 mL syringe with 18-gauge needle and incubated at 4°C for 30 min. Cell debris was removed by centrifugation at 17,000xg for 10 min at 4°C and 300 µL of dilution buffer (10 mM Tris/Cl pH 7.5, 150 mM NaCl, and 0.5 mM EDTA) supplemented with 1X protease and phosphatase inhibitors was added to the supernatant. 50 μL lysate was saved as input control. Lysates containing equal amounts of protein were incubated with 25 µL of GFP Trap beads slurry for 2 h at 4°C with gentle rotation. Post incubation, GFP Trap beads were sedimented/collected by centrifugation at 2500 *g* for 5 min, 4°C. Beads were washed thrice with 500 µL of wash buffer (10 mM Tris/Cl pH 7.5, 150 mM NaCl, 0.5% NP40, and 0.5 mM EDTA) and finally protein was eluted by boiling beads in 2X Laemmli buffer. Immunoprecipitated samples were analysed by Western blotting.

## Microscopy

All cells for imaging were grown on acid-washed coverslips (15 mm) placed in 24 well dishes and processed as per individual experiments. Details of cell treatment and fixation are given in respective sections below. Confocal microscopes used for each experiment are noted in the table below along with image aquisition parameters.

### Oil red O and BODIPY staining

Neutral lipids and lipid droplet (LD) morphology was studied by oil red O (ORO) staining (Mehlem et al., 2013). Cells grown on coverslips were treated with exosomes and fixed in 4% PFA for 15 min. Filtered working ORO solution (0.5% in isopropanol) was added for 15 min, nuclei counterstained with Hoechst 33,342 (Thermo-Fischer), and examined in brightfield at 100X as well as in fluorescence using a Texas red excitation filter (540–580 nm) and UV excitation filter (340–380 nm) on a confocal microscope (Olympus FV3000). Images were processed and analysed using ImageJ. Membranes enriched in ceramides and sphingolipids were visualized by incubating live cells for 30 min at 37°C with red fluorescent BODIPY-ceramide dye (10 µM, final concentration). Cells were fixed with 4% PFA for 20 min, washed with PBS, permeabilized with 0.2% Triton-X 100 for 10 min, washed again with PBS, nuclei counterstained with Hoechst 33,342, and visualised under a Zeiss LSM880 with Airyscan in Super Resolution Mode (zoom: 3.8 of 100X) using excitation/emission wavelength of 589/616 nm (Software: ZEN 2.3 SP1).

**Table:** 

	AiryScan Supplementary Figure S4C	Figure 6E	Figure 7G	Figure 7C	Supplementary Figure S4A
Pixel Format	2048 × 2048	1024 × 1024	1024 × 1024	1024 × 1024	1024 × 1024
Pinhole size	1.69	1.69	1	1	1.69
Frame averaging	2	2	2	2	2
Objective lens magnification (N.A.)	60X (1.42)	60X (1.42)	20X (0.75)	10X (0.4)	60X (1.42)
Laser line employed	405, 561	405,488,561	405, 640	405, 640	405, 640
Confocal microscope model	Zeiss LSM 880	Olympus FV3000	Olympus FV3000	Olympus FV3000	Olympus FV3000

#### Exosome labelling by PKH26

The optimum concentration of dye and exosomes for efficient PKH labelling were standardised for our system as recommended (Dehghani et al., 2020). Prior to staining, 10 µm PKH26 in 100 µL diluent C (PKH26GL, Sigma) was incubated at 37°C for 15 min. Then, appropriate volume of exosomes from the stock to provide 25 µg of exosomes in 100 µL of diluent was added to the dye + diluent, mixed gently and incubated at RT for 5 min. Excess dye was neutralised with 100 μL of EPM (contains 20% exosome-depleted FBS). Following dilution to 2 mL with DPBS, exosomes were transferred to Exosome Spin Columns (MW 3000, Invitrogen), and centrifuged at 4,000 *g* for 3 min at 4°C to remove unlabelled excess dye (used as a negative control) as per the manufacturer’s protocol. For the control sample, DPBS was used as the input replacing exosomes.

#### Exosome uptake inhibitor studies

Target cells cultured on coverslips under different conditions (MB, G_0_, R24 and MT) were pre-treated with inhibitors [Heparin (20 µg), Wortmannin (0.5 µm) or Dynole (10 µM)] in EPM for 30 min, then co-incubated with PKH26 labelled exosomes (25 μg/mL, refer Supplementary Material) for 4 h, following which cells were thoroughly washed with PBS and fixed with 4% PFA for 5 min, permeabilized with 0.5% Triton X-100, blocked with 10% FBS in PBS for 20 min and counterstained to visualize actin fibers [Alexa 488 Phalloidin (1:100)] and nuclei [DAPI (1 μg/mL)]. Immunofluorescence was analysed by confocal microscopy (Olympus FV3000) where PKH26 (Excited at λ_ex_ 551 nm, Emission ∼ λ_em_ 567 nm). Settings were kept constant for cells loaded with PKH26-labeled exosomes with or without with inhibitors. *Z*-stacked confocal images of phalloidin-stained cells were acquired to assess intensity of intracellular fluorescent particles. Image intensity was calculated using Fiji (ImageJ) software, and corrected mean intensity [CMI = Integrated density—(area of signal × mean background signal)] was determined for areas around at least 100 nuclei per sample.

### Immunofluorescence analysis

Cells were grown on glass coverslips, fixed with 2% PFA for 20 min at RT, permeabilized for 15 min with 0.5% Triton X-100 in PBS, blocked with 2% horse serum in 0.5% Triton X-100–PBS for 1 h, and incubated with primary antibody diluted in blocking buffer at RT for 1 h or overnight at 4°C. Antibody dilutions are given in Supplementary Table 2. The cells were then incubated in the appropriate secondary antibody for 45 min at RT. Nuclei were counterstained with DAPI (1 μg/mL) in PBS for 15 min. Samples were imaged using a confocal microscope (Olympus FV3000). Image intensity was calculated using Fiji (ImageJ) software, and corrected mean intensity (CMI = total intensity of signal—area of signal × mean background signal) was determined for more than 100 nuclei per sample.

### Plasmid construction and transfections

To prepare mCherry- and Flag-tagged Rab27a and GFP-tagged Kibra, gene-specific primers (with indicated restriction enzyme site adaptors) were used to amplify full length murine Kibra (3,312 bp) and Rab27a (665 bp) using cDNA from C2C12 and cloned into eGFP-N3 (XhoI, KpnI sites) and pmCherry-C1 (EcoRI, BamHI sites) respectively. CD63-GFP (CYTO120-PA-1) and CD9-RFP (CYTO123-PA-1) plasmids were purchased from System Biosciences (SBI); YFP-Endo plasmid encoding the endosome marker RhoB (Ghoshal et al., 2021) was a kind gift from Suvendra Nath Bhattacharyya. Promoter-luciferase constructs for Myogenin (1621bp) and MCK (3,357 bp) were prepared by amplification from the genomic DNA of C2C12 and cloned into pGL3-basic vector (E1751, Promega) (Primer sequences are given in Supplementary Table 1, Supplementary Material). C2C12 myoblasts plated on cover slips (for imaging) or 12-well plates (for luciferase assays) or 60 mm dishes (for immunoblotting) were transfected as previously described using Lipofectamine LTX reagent (15338100, Invitrogen) (Sebastian et al., 2009). For signaling assays, cells were treated with exosomes (25 μg/mL) for 24 h by addition to the growth medium.

### TOP/FOPflash reporter assay

Stable cell lines derived from single clones of TOPflash or FOPflash transfected in C2C12 cells (Subramaniam et al., 2014) were used for testing Wnt-mediated transcriptional activity of exosomes derived from different cellular states. TOP-Flash construct (Tcf/Lef reporter Super 8x), (Veeman et al., 2003), contains multimerized Tcf/Lef-binding sites; specificity is provided by the control FOP-Flash which contains mutated binding sites. For reporter analysis, target cells in 24-well plates at 70% confluency (both TOP and FOP stable cells) were treated with different donor exosomes (25 μg/mL) or Wnt activator, CHIR99021 (3 and 5 μM) for 24 h. Dual luciferase assay was performed as per manufacturer’s protocols (Roche). BCA protein quantification was performed and relative light units (RLU) measured in a luminometer (Enspire, Perkin Elmer) was normalized to total protein in the lysate (RLU per μg protein). PBS without exosomes was used as a negative control, while FOP-flash cells served as control for specificity of Wnt-activated luciferase.

### Cell proliferation assay (EdU incorporation)

C2C12 cells plated on cover slips were cultured under different conditions [MB or G_0_] were pulsed with 10 µm EdU (5-ethynyl-2′-deoxyuridine) for 30 min prior to fixation with 2% PFA for 20 min, permeabilised and blocked (0.5% Triton X-100 + 10% FBS in PBS). Click-iT EdU reaction cocktail was used as per the manufacturer’s instructions to detect EdU-positive S-phase nuclei and counterstained with DAPI to visualise all nuclei using a Zeiss Axio Imager two epi-fluorescence microscope. No EdU control was negative and no cross reactivity of secondary reagents was detected.

### Colony formation assays

MB cells treated with exosomes derived from different donor cells MB, G_0_, R24 and MT (25 µg) for 24 h, were plated at clonal density (500 cells/150 mm dish) and cultured for 7 days. Colonies were stained with methylene blue for counting.

### Image analysis and statistics

For all experiments unless otherwise mentioned, three biological replicates were used. Statistical analysis values are presented as mean ± SEM. Statistical differences between means were determined using the unpaired/paired Student’s *t-*test or one-way ANOVA as indicated. All analyses were computed using GraphPad Prism six software. Differences were considered as statistically significant for *p* < 0.05. Digital images were captured and processed using ImageJ (Fuji); composites and overlays were prepared using ImageJ; minimal adjustments to brightness or contrast were uniformly applied to entire image. The spatial colocalization between different proteins of interest each labelled with a different fluorophore was measured by calculating Pearson’s correlation coefficient (*r*) using the JACoP plug-in of Fiji (ImageJ) software.

## Data Availability

The datasets presented in this study can be found in online repositories. The names of the repository/repositories and accession number(s) can be found below: https://www.ebi.ac.uk/pride/archive/, PXD045797.
