# Supplementary material for: Enhanced secretion of promyogenic exosomes by quiescent muscle cells
Source: Front Cell Dev Biol. 2024 Jul 23;12:1381357. doi: 10.3389/fcell.2024.1381357 (PMC11301339; doi:10.3389/fcell.2024.1381357)
Supplement: Supplementary file 1 [file DataSheet1.PDF]

Figure S1

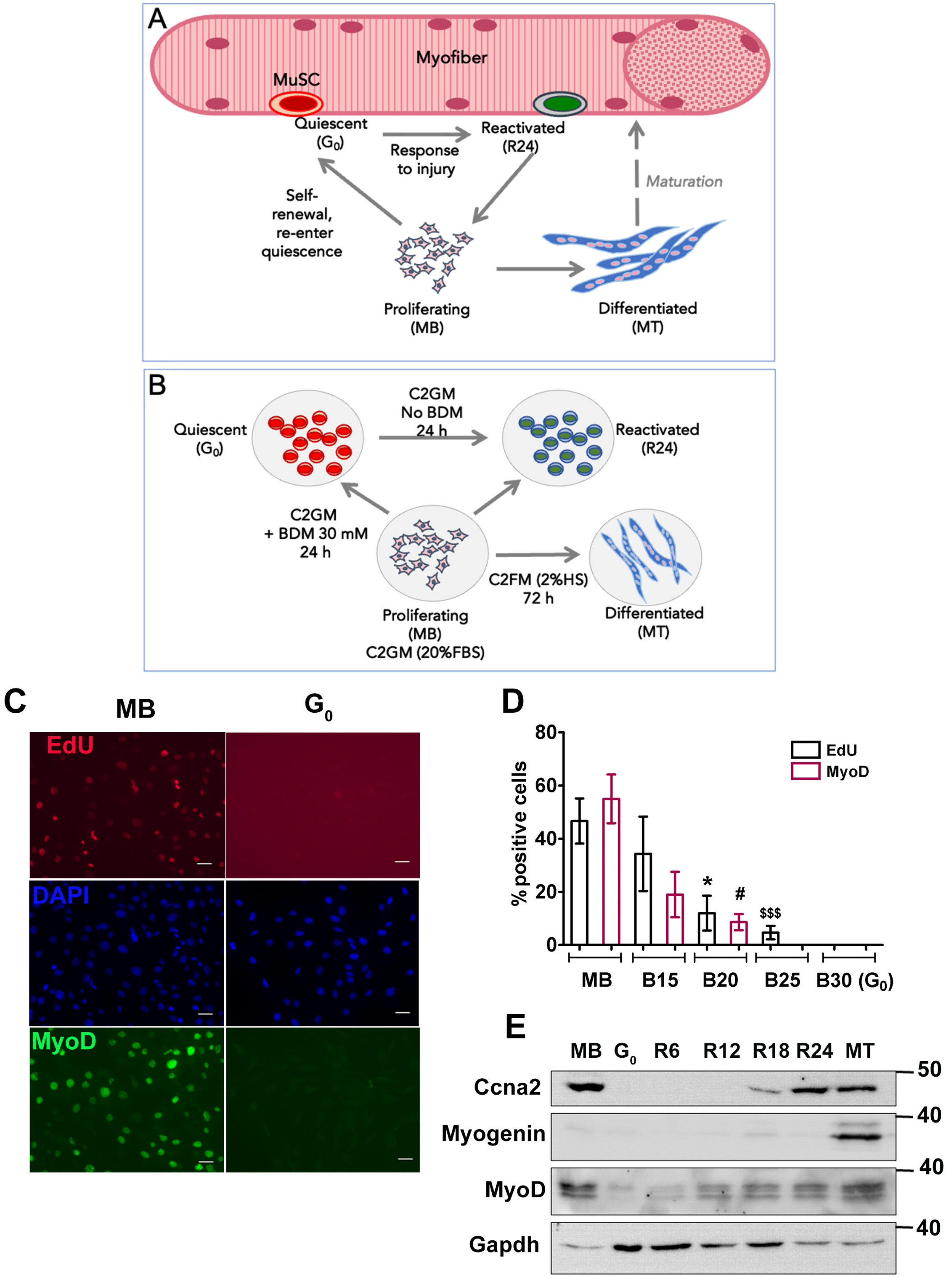
A
Myofiber
MuSC
Quiescent (G0)
Response to injury
Reactivated (R24)
Maturation
Self-renewal, re-enter quiescence
Proliferating (MB)
Differentiated (MT)
B
Quiescent (G0)
C2GM No BDM 24 h
Reactivated (R24)
C2GM + BDM 30 mM 24 h
C2FM (2%HS) 72 h
Proliferating (MB) C2GM (20%FBS)
Differentiated (MT)
C
MB
G0
EdU
DAPI
MyoD
D
% positive cells
80
60
40
20
0
EdU
MyoD
*
#
$$$
MB
B15
B20
B25
B30 (G0)
E
MB
G0
R6
R12
R18
R24
MT
Ccna2
Myogenin
MyoD
Gapdh
50
40
40
40

Figure S2

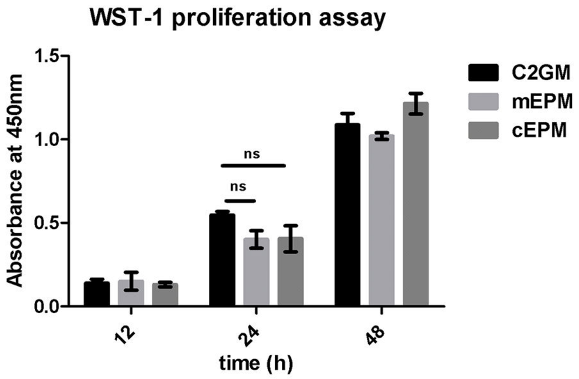
WST-1 proliferation assay
Absorbance at 450nm
1.5
1.0
0.5
0.0
ns
ns
12
24
48
time (h)
C2GM
mEPM
cEPM

Figure S3

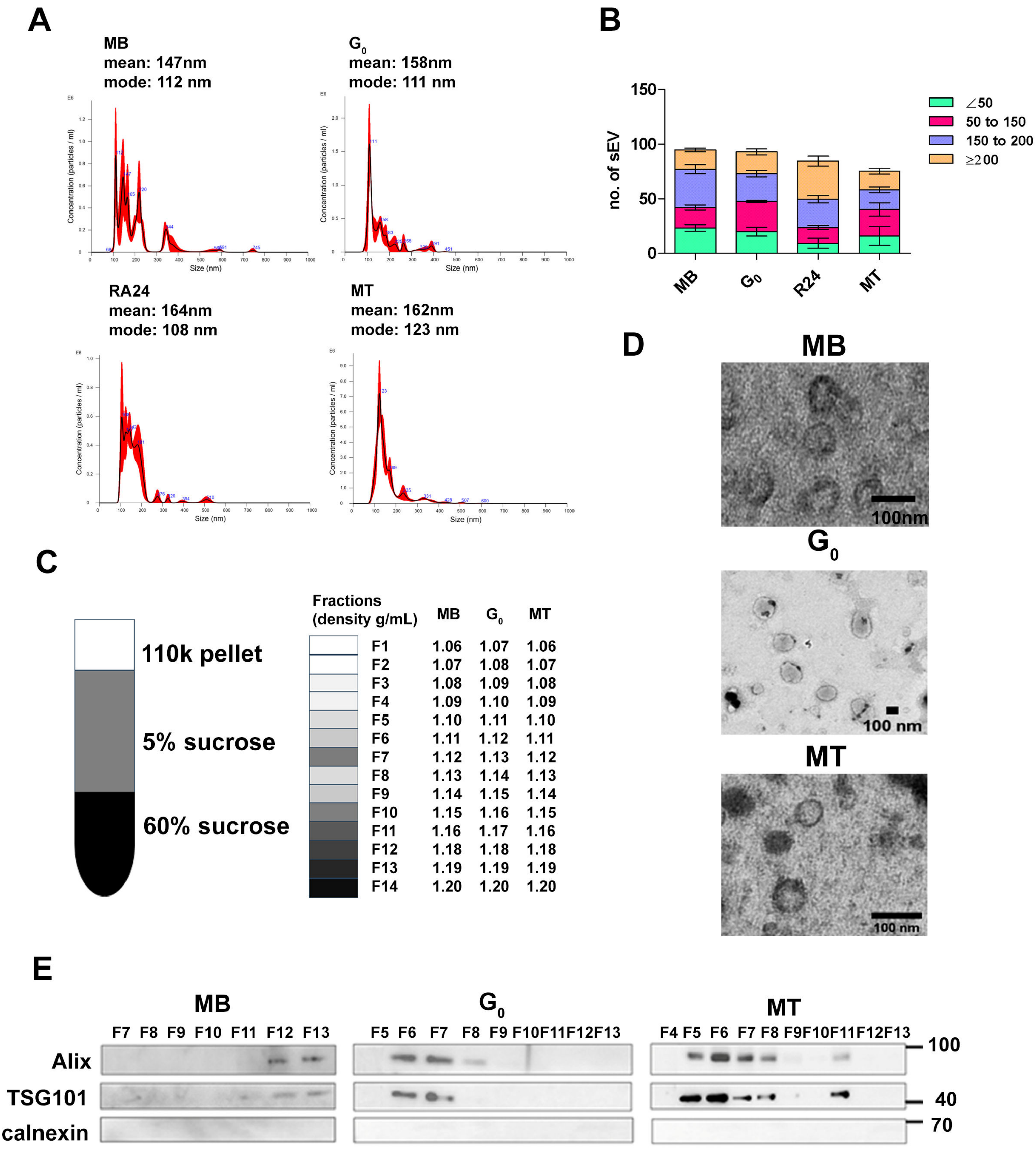
A
MB
mean: 147nm
mode: 112 nm
G0
mean: 158nm
mode: 111 nm
RA24
mean: 164nm
mode: 108 nm
MT
mean: 162nm
mode: 123 nm
Concentration (particles / ml)
Size (nm)
B
no. of sEV
∠50
50 to 150
150 to 200
≥200
MB
G0
R24
MT
C
110k pellet
5% sucrose
60% sucrose
Fractions (density g/mL)
MB G0 MT
F1 1.06 1.07 1.06
F2 1.07 1.08 1.07
F3 1.08 1.09 1.08
F4 1.09 1.10 1.09
F5 1.10 1.11 1.10
F6 1.11 1.12 1.11
F7 1.12 1.13 1.12
F8 1.13 1.14 1.13
F9 1.14 1.15 1.14
F10 1.15 1.16 1.15
F11 1.16 1.17 1.16
F12 1.18 1.18 1.18
F13 1.19 1.19 1.19
F14 1.20 1.20 1.20
D
MB
100nm
G0
100 nm
MT
100 nm
E
MB
F7 F8 F9 F10 F11 F12 F13
G0
F5 F6 F7 F8 F9 F10 F11 F12 F13
MT
F4 F5 F6 F7 F8 F9 F10 F11 F12 F13
Alix
TSG101
calnexin
100
40
70

Figure S4

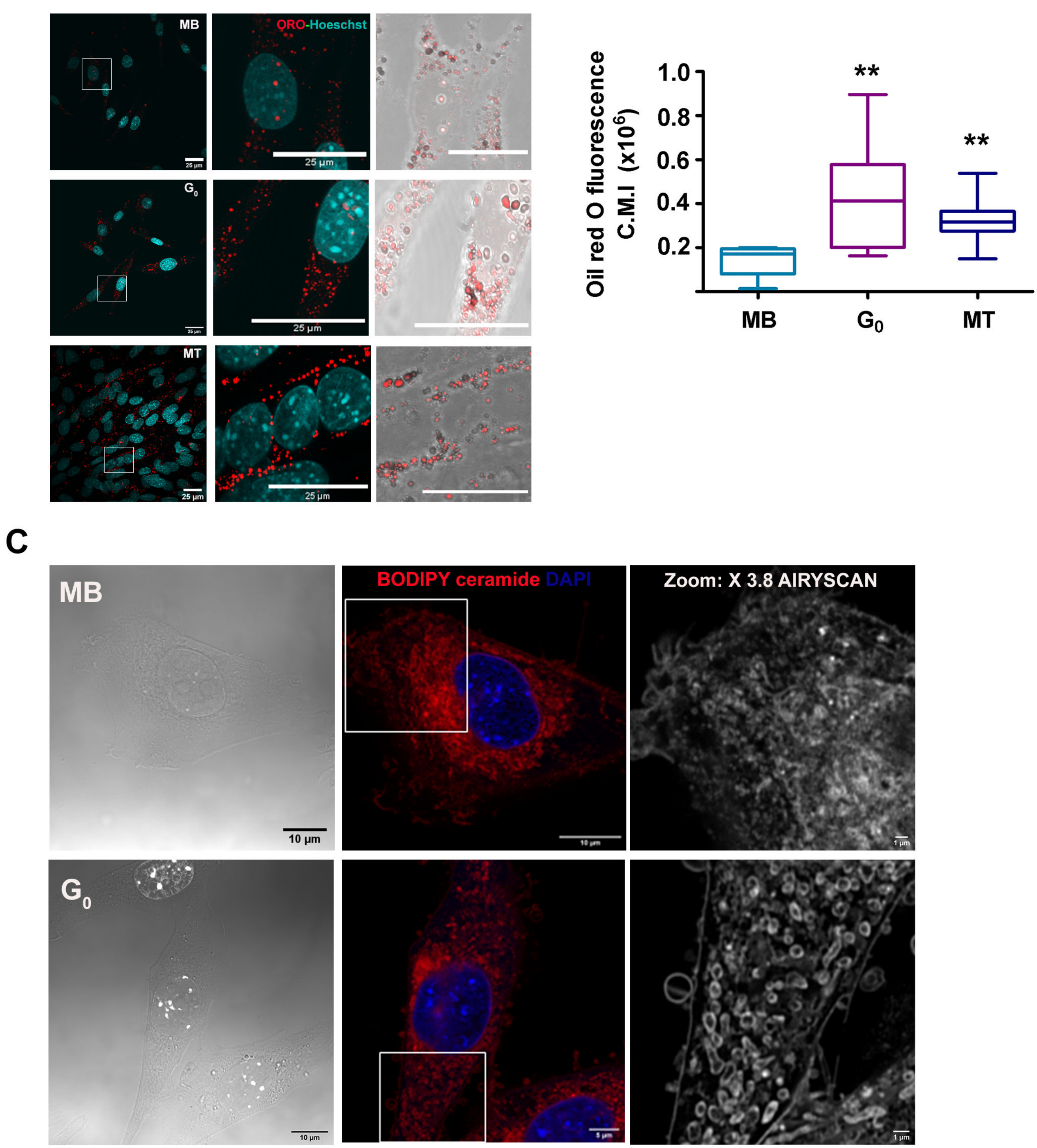
A
MB
ORO-Hoeschst
25 µm
G0
MT
B
Oil red O fluorescence C.M.I (x10^6)
1.0
0.8
0.6
0.4
0.2
**
**
MB
G0
MT
C
MB
BODIPY ceramide DAPI
Zoom: X 3.8 AIRYSCAN
10 µm
1 µm
G0
5 µm

Figure S5

A

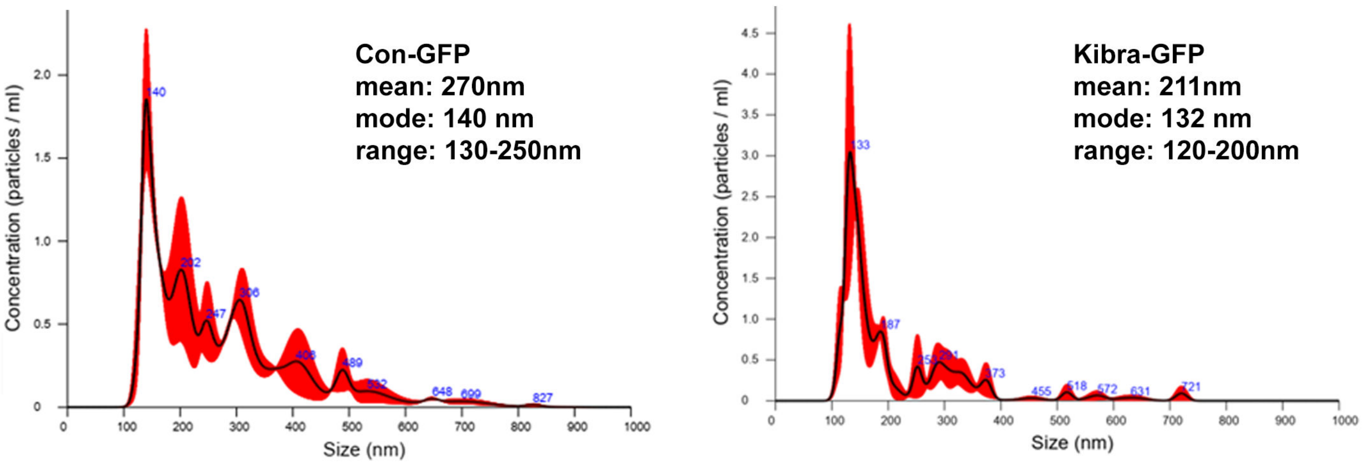
Con-GFP
mean: 270nm
mode: 140 nm
range: 130-250nm
Kibra-GFP
mean: 211nm
mode: 132 nm
range: 120-200nm
Concentration (particles / ml)
Size (nm)

B

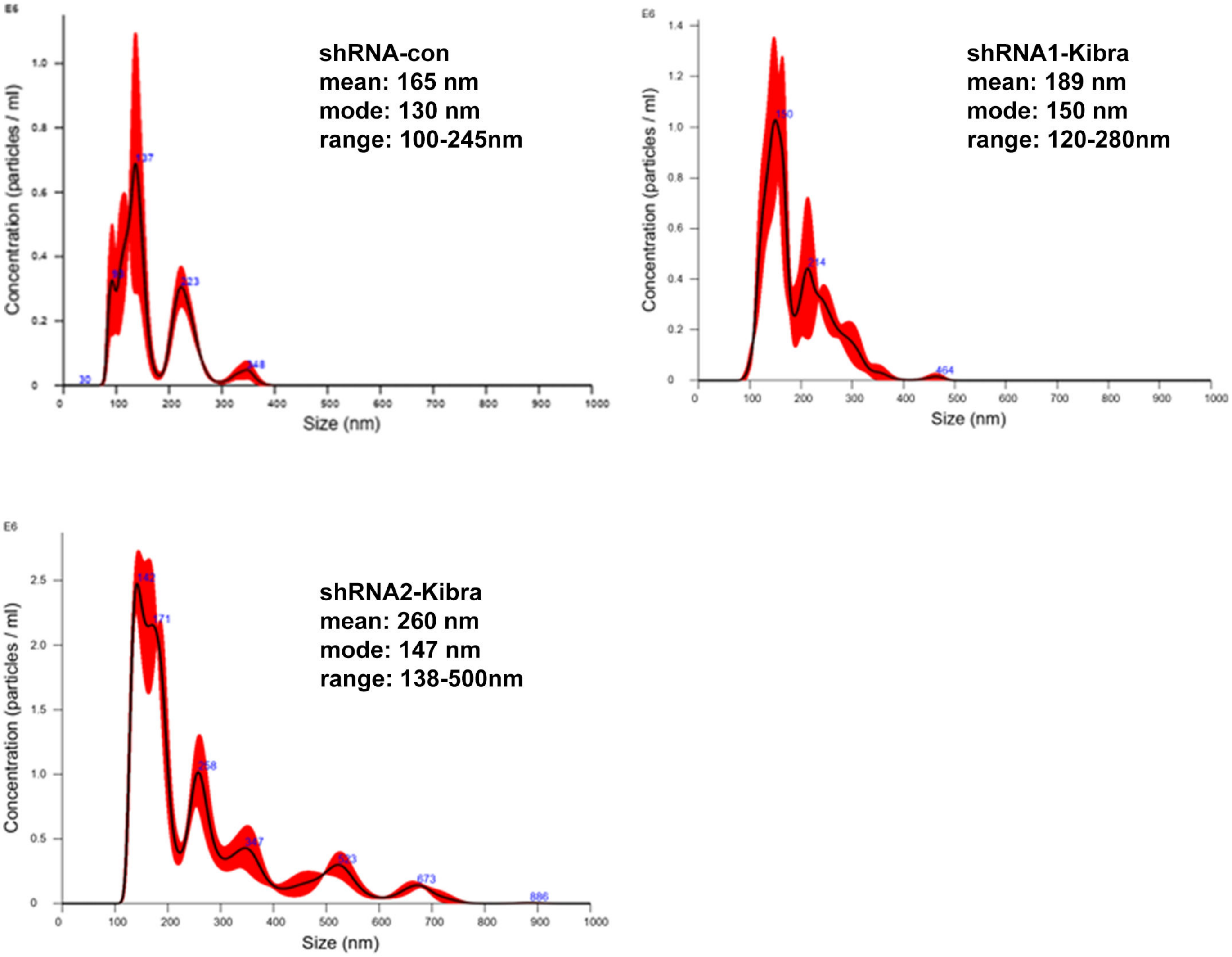
shRNA-con
mean: 165 nm
mode: 130 nm
range: 100-245nm
shRNA1-Kibra
mean: 189 nm
mode: 150 nm
range: 120-280nm
shRNA2-Kibra
mean: 260 nm
mode: 147 nm
range: 138-500nm
E6
Concentration (particles / ml)
Size (nm)

Figure S6

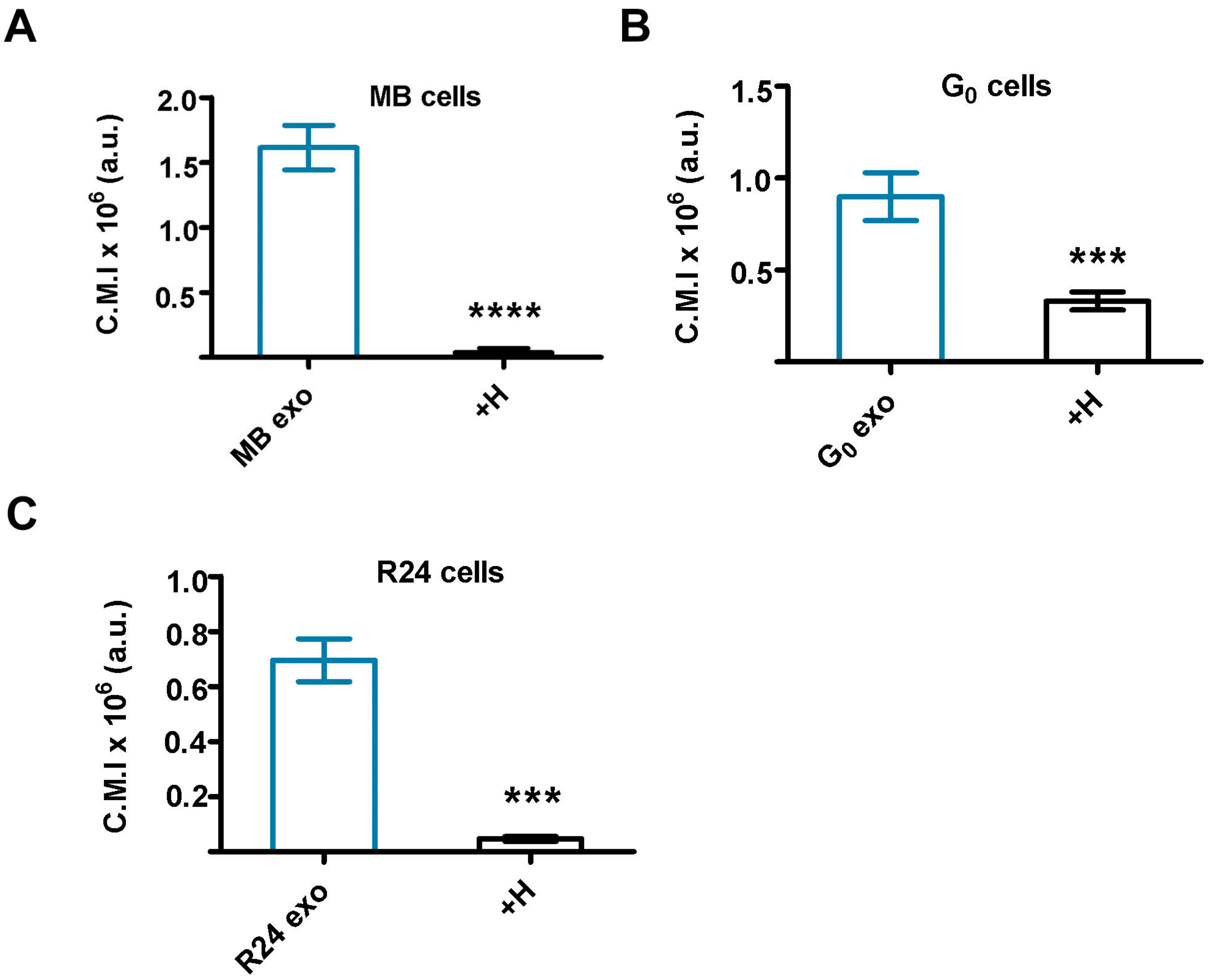
A
MB cells
C.M.I x 10^6 (a.u.)
2.0
1.5
1.0
0.5
****
MB exo
+H
B
G_0 cells
C.M.I x 10^6 (a.u.)
1.5
1.0
0.5
***
G_0 exo
+H
C
R24 cells
C.M.I x 10^6 (a.u.)
1.0
0.8
0.6
0.4
0.2
***
R24 exo
+H

Figure S7

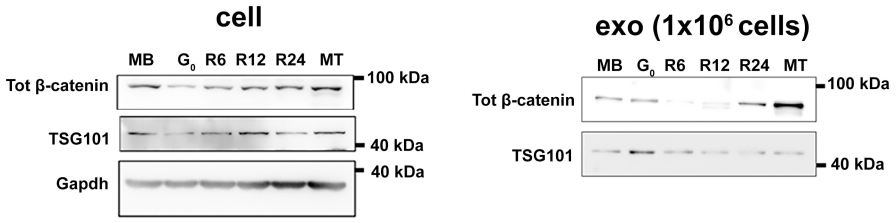

cell
MB
G0
R6
R12
R24
MT
Tot β-catenin
TSG101
Gapdh
100 kDa
40 kDa
40 kDa
exo (1x10^6 cells)
MB
G0
R6
R12
R24
MT
Tot β-catenin
TSG101
100 kDa
40 kDa

Supplementary Information

Figure legends

Fig. S1. ***Cellular states of skeletal muscle cells in vivo can be recapitulated in vitro.***

A. Schematic showing muscle cells in different states in adult skeletal muscle tissue. Quiescent MuSc (G_0) are activated to enter cell cycle (R24) which then undergo clonal expansion to become proliferating MB. Under differentiation cues, MB fuse to form multinucleated MT which mature to become myofibers, and recreate a niche for a few MuSC to return to G_0 and repopulate the stem cell pool. **B**. Schematic showing the generation of muscle cells states generated *in vitro* using cultured mouse myoblasts to recapitulate physiological *in vivo* states: Sub-confluent cultures of asynchronously proliferating myoblasts (MB) are treated with BDM (myosin II inhibitor) for 24 hours in growth medium (GM, 20% FBS) to generate synchronized cultures of quiescent myoblasts (G_0). After 24 hours, GM+BDM is removed and replaced with fresh GM (without BDM) for 24 hours to generate synchronously reactivated myoblasts (R24). To generate differentiated myotubes (MT), dense cultures of MB (90% confluency) are incubated in differentiation medium (DM, 2% HS) for 3 days during which time they fuse to form multinucleated myotubes (MT). **C**. IFA analysis showing the absence of both EdU incorporation (proliferation) and MyoD expression (lineage determinant) in G_0, as is typical of MuSC *in vivo*. **D**. Dose-dependent inhibition of proliferation (EdU incorporation) by BDM treatment. **E.** Immunoblot analysis of cell cycle and myogenic regulatory proteins in MB, G_0, during 6-24 hr after reactivation from G_0 (R6 to R24) and MT. Note that the distinct combinations of cyclin A2, Myogenin and MyoD molecularly define the key characteristics of all 4 states.

Fig S2. ***Test of exosome production medium for proliferation-promoting activity***.

WST-1 cell proliferation assay confirmed that exosome production medium (EPM) does not affect myoblast proliferation over 48 hours. EPM is prepared by depleting serum exosomes from growth media so that they do not co-purify with cell-derived exosomes and confound the analysis. Cells which had been cultured to achieve different states were incubated for an adidtional 6 hrs in manually prepared EPM (mEPM) or commercial EPM (cEPM), exposed to WST and cell number determined by absorbance at 450 nm. Values represent 3 independent experiments, ($p=0.06$ or 0.07, non-significant). All exosome harvesting experiments were performed by

Fig. S3 ***Biophysical features of sEVs from muscle cells are consistent with exosomes.***

A. NTA traces showing concentration of particles/ml with size distribution (diameter in nm) from muscle cells cultured in conditions of proliferation (MB), quiescence (G_0), reactivation for 24 hrs (R24) and 3 days of differentiation (MT). Inset values show mean and mode. Exosomes were isolated from 6 hrs CM from equal numbers of cells (10^6) in each state.

B. Quantification of particle size derived from TEM images of purified exosomes from MB, G_0, R24 and MT, n=2. At least 100 particles were counted from images of each condition and diameter estimated using ImageJ. Graph indicates size range (nm) plotted against number of particles in each bin. The bulk of particles in each condition fell in the range 50-150 nm.

C. Sucrose gradient flotation of exosomes from different cellular states is consistent with heterogeneity observed in NTA and TEM analysis. Pellets obtained after 110, 000xg centrifugation floated on an underlying sucrose gradient (60% to 5%). A total of 14 fractions were collected, washed, recentrifuged at 100,000xg to collect exosomes in each fraction.

D. TEM imaging of whole mounts of exosomes negatively stained with uranyl acetate and embedded in methyl cellulose**,** from fraction #6 (G_0 and MT) and fraction #12 (MB), n=2, scale bar: 100 nm. All images showed the presence of intact vesicles of heterogenous size.

E. Western blotting of sucrose gradient fractions for Alix and TSG101 confirms the endosomal origin of the sEVs, and absence of calnexin, an ER marker confirms the purity. Density of each fraction was measured by correlating with refractive index and is indicated over the respective lane (n=2).

Fig. S4. G_0 cells exhibit increased accumulation of lipid droplets (LD) and ceramide-rich vesicles. **A**. Confocal imaging of MB, G_0 and MT stained with Oil Red O (ORO); nuclei are stained with Hoechst 33342. Left panels show cells at 40x magnification. Middle and right-hand panels show zoomed images, scale bar represents 25 µm. ORO-stained lipid droplets are seen under fluorescence (middle) and brightfield. Scale bar represents 25 µm. **B**. Quantitative measurement of ORO fluorescence around at least 100 nuclei in each condition represented as corrected mean intensity (C.M.I) from three independent experiments. Data is represented from three independent experiments as mean ± SE, * $P \leq 0.05$, and *** $P \leq 0.0005$. **C.** Visualization of vesicles rich in ceramide in G_0 cells. Representative confocal images of BODIPY TR Ceramide staining of MB and G_0 cells. Scale bar represents 10 µm. The zoomed images (x 3.8) of the boxed region represent Airyscan imaging (super-resolution) of stained sphingolipid rich vesicular structures taken on a Zeiss LSM880 microscope. Scale bar represents 1 µm.

Fig. S5. NTA analysis of exosome secretion by Kibra over-expressing (Kibra-GFP) and Kibra knockdown (shRNA) cells.

Representative NTA traces showing concentration of particles/ml with size distribution (diameter in nm) from muscle cells cultured in conditions of **A.** Kibra-GFP overexpression and **B.** Kibra knockdown with two distinct shRNAs cells. Inset values show mean and mode. Exosomes were isolated from 6 hrs CM from equal numbers of cells (10^6) in each state. Quantification of the concentration is shown in the main Fig 3.

Fig. S6. Uptake of self-derived exosomes by target cells is via a heparin-sensitive pathway suggesting HSPG involvement. Exosomes (25 µg) derived from three different donor cell states (MB, G_0 and R24) were added to target cells in the same state (MB/G_0/R24 respectively) in absence (exo) or presence of Heparin (+H, 20 µg/ml) for 4 hrs. Quantitative analysis for PKH26 fluorescence was done by confocal imaging (n=3), *** indicates $p \leq 0.0005$, in comparison to untreated control.

Fig. S7. Wnt effector β-catenin protein in exosome fractions derived from different cell states

Immunoblot analysis of total β-catenin in MB, G_0, during 6, 12-24 hr after reactivation from G_0 (R6 to R24) and MT. Differential expression was observed in cell lysates (20 µg) and in exosome fractions (exo from $1x10^6$ cells); MT show the greatest enrichment.

Tables

Table 1 lists the sequences of shRNAs and primers used for cloning.

Table 2 lists the antibodies used in Immunoblotting and Immunofluorescence analysis

Devan et al.

Table 1

Sl. No.	Plasmid Name	Primer Name	Primer Sequence (5'-3')
1	Kibra-GFP	KIBRA-XhoI-Fwd	TATATACTCGAGATGCCCCGGCCGGAGTTG
2	Kibra-GFP	KIBRA-KpnI-Rev	TATATAGGTACCGACGTCATCTGCAGAGAGAGCTGGGATG
3	Rab27a-mCherry	Rab27a-EcoRI-Fwd	TATATAGAATTCTATGTCGGATGGAGATTACGATTACCTCATCAA
4	Rab27a-mCherry	Rab27a-BamHI-Rev	TATATAGGATCCAACCAGGGGTCATGCGCTG
5	Kibra-shRNA1	shKIBRA-Fwd-2	CCGGGATTACTTCATAGACCACAATCTGCAGATTGTGGTCTATGAAGTAATCTTTTTG
6	Kibra-shRNA1	ShKIBRA-Rev-2	AATTCAAAAAGATTACTTCATAGACCACAATCTGCAGATTGTGGTCTATGAAGTAATC
7	Kibra-shRNA2	shKIBRA-Fwd-3	CCGGGAGATCCTGAAAGCTGAAATTCTGCAGAATTTCAGCTTTCAGGATCTCTTTTTG
8	Kibra-shRNA2	shKIBRA-Rev-3	AATTCAAAAAGAGATCCTGAAAGCTGAAATTCTGCAGAATTTCAGCTTTCAGGATCTC
9	Control-shRNA	Con-shRNA-Fwd	CCGGCAACAAGATGAAGAGCACCAACTCGAGTTGGTGCTCTTCATCTTGTTGTTTTTG
10	Control-shRNA	Con-shRNA-Rev	AATTCAAAAACAACAAGATGAAGAGCACCAACTCGAGTTGGTGCTCTTCATCTTGTTG
11	MCK-luc	MCK-Promoter_KpnI-Fwd	TATATAGGGGTACCCCATCCTGGTCTATAGAGAGAGT
12	MCK-luc	MCK-Promoter-XhoI-Rev	TATATATACTCGAGGTGACCCGGGGGCAGC
13	Myogenin-luc	MyoG-KpnI-F	TATAGGTACCTCTAGAGTTGTATGACGCAGGC
14	Myogenin-luc	MyoG-XhoI-R	TATACTCGAGCATCAGGTCGGAAAAGGCTT

Table 2

Antibody	Dilution (IF)	Dilution (Western blot)	Type	Species	Cat. number	Company
Alix	-	1:1000	Monoclonal	Mouse	ab117600	Abcam
Calnexin	-	1:5000	Monoclonal	Rabbit	C4731	Sigma
CD9	-	1:1000	Monoclonal	Rabbit	EXOAB-CD9A-1	System Biosciences
Cyclin A2	-	1:2000	Monoclonal	Rabbit	Ab181591	Abcam
Eea1	-	1:1000	Monoclonal	Rabbit	#3288	CST
Flag	-	1:1000	Monoclonal	Rabbit	#14793	CST
Flotillin-1	-	1:2000	Polyclonal	Rabbit	F1180	Sigma
Gapdh	-	1:10000	Monoclonal	Mouse	Ab8245	Abcam
GFP	-	1:1000	Monoclonal	Rabbit	2956S	CST
Hsp70	-	1:1000	Monoclonal	Mouse	ADI-SPA-820	Enzo Life Sciences
Hsp90	-	1:5000	Monoclonal	Mouse	sc-13119	Santa Cruz
Kibra	-	1:1000	Polyclonal	Rabbit	#8774	CST
MyoD	1:200	1:500	Monoclonal	Mouse	M3512	Dako
Myogenin	1:200	1:500	Monoclonal	Mouse	sc-12732	Santa Cruz
MyHC	1:1	1:1	Monoclonal	Mouse	A4.1025	Hybridoma (Hughes et al. 1993; Webster et al. 1988)
P130	-	1:1000	Polyclonal	Mouse	sc-317	Santa Cruz
Qsox1	-	1:1000	Polyclonal	Rabbit	12713-1-AP	Proteintech
Rab27a	1:200	1:2000	Polyclonal	Rabbit	17817-1-AP	Proteintech
TSG101	-	1:2000	Polyclonal	Rabbit	T5701	Sigma

Total β-Catenin	-	1:1000	Polyclonal	Rabbit	C2206	Sigma
HRP conjugated Goat Anti-Mouse IgG	-	1:5000	Polyclonal	Goat	115-035-166	Jackson ImmunoResearch
HRP conjugated Goat Anti-Rabbit IgG	-	1:5000	Polyclonal	Goat	711-035-152	Jackson ImmunoResearch
Donkey anti-Rabbit Alexa fluor 568	1:500	-	Polyclonal	-	A-10042	Thermofisher Scientific
Donkey anti-Mouse Alexa fluor 647	1:500	-	Polyclonal	-	A-31571	Thermofisher Scientific
